# Sex disparities revealed by single-cell and bulk sequencing and their impacts on the efficacy of immunotherapy in esophageal cancer

**DOI:** 10.1186/s13293-024-00598-z

**Published:** 2024-03-15

**Authors:** Huimeng Yan, Jinyuan Huang, Yingying Li, Bin Zhao

**Affiliations:** 1 Second Affiliated Hospital, Yuying Children’s Hospital, Wenzhou Medical University, Wenzhou, 325035 China; 2 Quanzhou First Hospital Affiliated to Fujian Medical University, Quanzhou, 362000 China

**Keywords:** Sex disparities, Immunotherapy, Esophageal cancer, T cell, scRNA-seq

## Abstract

**Background:**

There is an ongoing debate on whether sex affects immune-suppressive tumor microenvironment and immunotherapy. Here, we explored the underlying molecular bases for sex dimorphisms and their impact on the efficacy of immunotherapy in esophageal cancer (EC).

**Methods:**

2360 EC patients from phase 3 trials were pooled to compare overall survivals by calculating hazard ratios (HRs) and their 95% confidence intervals (CIs). Genomic data of 1425 samples were integrated to depict the genomic landscapes and antigenic features. We also examined the sex disparities based on single-cell RNA sequencing and T cell receptor-sequencing data from 105,145 immune cells in 60 patients.

**Results:**

Immunotherapy was associated with favorable outcomes in men (HR, 0.71; 95% CI, 0.65–0.79; *P* < 0.001), but not in women (HR, 0.98; 95% CI, 0.78–1.23; *P* = 0.84) (*P*_interaction_ =0.02). The frequencies of 8 gene mutations, 12 single base substitutions signatures, and 131 reactome pathways were significantly different between male and female. Additionally, six subtypes of HLA-II antigens were enriched in women. Hence, we constructed and then validated a sex-related signature to better predict the outcomes of immunotherapy. Exhausted CD8^+^ T cells were highly infiltrated in men, while naïve CD8^+^ T cells were more common in women. Further examinations on multiple malignancies suggested exhausted CD8^+^ T cells were enriched in patients who responded to immunotherapy.

**Conclusions:**

Our study delineated the robust genomic and cellular sex disparities in EC. Furthermore, male, rather than female, derived significantly benefits from immunotherapy. These results have implications for treatment decision-making and developing immunotherapy for personalized care.

**Plain English Summary:**

In the past several years, immunotherapy has gradually replaced the traditional chemotherapy as the standard treatment in esophageal cancer. It is well-established that immunological responses in male and female differ significantly. However, there is an ongoing debate on whether sex can impact the treatment outcomes in immunotherapy. In the present study, we systematically characterized the genomic and cellular landscapes of esophageal cancer, and revealed the significant differences between male and female patients. Furthermore, with over 2000 patients with esophageal cancer, we showed that only men can benefit from immunotherapy. In women, immunotherapy failed to show superior over chemotherapy. These results have implications for treatment decision-making and developing next-generation immunotherapy for personalized care.

**Supplementary Information:**

The online version contains supplementary material available at 10.1186/s13293-024-00598-z.

## Highlights

**•** In esophageal cancer (EC), immunotherapy was associated with favorable outcomes in men, but not in women.
**•** The frequencies of 8 mutant genes, 12 SBS, and 131 reactome pathways were significantly different between male and female.
**•** We developed a sex-related signature to predict the outcomes of EC immunotherapy.
**•** Exhausted CD8^+^ T cells were highly infiltrated in male EC patients, while naïve CD8^+^ T cells were more common in female patients.

**Supplementary Information:**

The online version contains supplementary material available at 10.1186/s13293-024-00598-z.

## Introduction

Esophageal cancer (EC) is one of the most common and fatal malignancies globally. It is estimated that the newly diagnosed cases and deaths associated with EC are 604,100 and 544,076 annually [1]. In the past several years, the therapeutic landscape for EC has changed dramatically due to the application of immunotherapy. Immune checkpoint inhibitors (ICIs) targeting cytotoxic T-lymphocyte-associated antigen 4 (CTLA-4), programmed cell death protein 1 (PD-1), and programmed cell death ligand 1 (PD-L1) can significantly improve the overall survival (OS) in many malignancies [2]. In EC, since the U.S. Food and Drug Administration (FDA) approved pembrolizumab for patients whose tumors express PD-L1 in 2019, immunotherapy has gradually replaced the traditional chemotherapy as the standard treatment [3].

It is well-established that immunological responses to both extrinsic and intrinsic-antigens differ significantly between male and female [4, 5]. In cancer, bioinformation analyses shows that, compared to men, women harbor greater intra-tumoral accumulation of activated T cells, as well as counter-balancing immune suppressor cells [6]. Unfortunately, although it is clear that sex can robustly affects immune regulation and responses, there is an ongoing debate how they contribute to the immune-suppressive tumor microenvironment and the response to immunotherapy [4]. Notably, a meta-analysis on 20 randomized controlled trials (RCTs) with 7 types of tumors revealed that women presented poor outcomes upon ICI-based treatment [7]. However, this result was soon challenged by Wallis et al. [8], who demonstrated there was no statistically significant association between sex and survival in caner immunotherapy. It may seem that sex-based dimorphism in the efficacy of immunotherapy is depended on tumor type. For example, women with advanced lung cancer show more survival benefit than man when treated with combination of immunotherapy and chemotherapy [9]. However, men had higher overall survival rates compared to women in melanoma [10–13]. In EC, it was well-known that the incidence rate was approximately 3-fold higher in male compared with female patients [1]. As for treatment outcomes, although sex-specific differences had been reported in several studies, most of these trials were not immunotherapy-related and too small to draw any solid conclusion [14]. Accordingly, more in-depth research on sex bias in the efficacy of EC immunotherapy were needed.

In the present study, we first conducted a meta-analysis on phase 3 studies to explore the impact of sex on survivals in patients treated with ICIs. Next, the whole-genome sequence (WGS) or whole-exome sequence (WES) of 1425 EC samples from 13 datasets were integrated to depict the genomic landscapes of EC, and to explore the antigenic features underlying male and female. Moreover, we constructed a novel sex-related signature (SRS) as predictive biomarker for the efficacy of immunotherapy. We also systematically examined the sex disparities in the compositions of various cell populations based on the scRNA-seq datasets. Our study may have implications in developing cancer immunotherapy for personalized and patient-centered care.

## Methods

### Meta-analysis on the efficacy of immunotherapy based on sex differences

A comprehensive search of Embase, PubMed and Cochrane databases for trials conducted in patients with EC from inception to September 2023 was carried out with no language restriction. Both inclusion and exclusion criteria were pre-specified. To be eligible, studies had to meet the following criteria: (1) study design: RCT irrespective of blindness and line of treatment; (2) population: adult patients with unresectable, advanced, recurrent, or metastatic EC; (3) intervention: treated with ICIs that were approved by FDA, namely atezolizumab, avelumab, cemiplimab, dostarlimab, durvalumab, ipilimumab, nivolumab, and pembrolizumab; (4) comparison: patients in control arms should be treated with conventional chemotherapy; (5) main outcomes: overall survival (OS) measured as hazard ratio (HR) and its corresponding 95% confidence interval (CI) according to patients’ sex subgroup. Studies were excluded if they were retrospective or prospective observational cohort studies. In addition, Phase 1 and non-randomized phase 2 studies were excluded. Other publications on the topic, including commentaries, review articles, conference abstract, quality of life studies, editorials, cost effectiveness analyses, were also not included. Any disagreements were resolved by discussion and consensus.

The five-point Jadad ranking system [15] was applied to access the risk of bias. We evaluated quality of double-blinding, randomization, withdraw and dropout of patients and scored each trial between zero (poor methodological quality) and five (optimal methodological quality).

The primary endpoint was the efficacies of immunotherapy in male and female, measured in terms of the hazard ratio for death in the intervention arm compared with those treated with chemotherapy. Statistical heterogeneity between different trials was assessed by Cochrane’s Q statistic. The *I*^*2*^ statistic was calculated to assess the extent of inconsistency contributable to the heterogeneity across different studies [16]. The assumption of homogeneity was considered invalid for *I*^*2*^ > 25% and *P* < 0.10. Hence, the fixed-effects models were utilized to estimate the size of the treatment benefit. Tests of interaction were calculated to evaluate the differences in treatment effect across subgroups.

### Data selection for WGS/WES analysis

We searched the genomic databases including EBI-ENA, NCBI-SRA, and NGDC-GSA, for all publicly available WGS/WES data. Additionally, potential papers in PubMed and Embase, and the references of relevant articles were examined. Moreover, the public cancer genome databases including ICGC, TCGA, and COSMIC mutation database were screened. When multiple publications of the same population appeared or if there was a case mix between different publications, only the most recent and/or most complete reporting study was included. Data were excluded if patients were diagnosed with multiple primary cancers or the esophageal tumors of uncertain pathological histophysiology. Information obtained from metastatic sites were also excluded. The available clinicopathological features including age, sex, tumor stage, drinking history, and tumor location were directly derived from the original reports. All vague or misleading information were treated as not available.

### WGS/WES raw sequence data processing, integration and annotation

For NCBI-SRA data, we used SRA-Tools to obtain fastq files, which were conducted quality control by fastp with default parameters [17]. The different SRA files from same samples were combined before mapping, which was performed by BWA to hg38.p13 genome. The bam files were de-duplicated and re-calibrated by GATK. The pairwise relationships between normal and tumor samples were investigated by BAM-matcher [18]. The insertion or deletion mutations (INDELs) and single nucleotide variants (SNVs) were examined by Mutect2. The enrolled patients and their genomic information were collected by their original names. We exclude all duplicated data in the final integration. The quality-controlled results were combined into a VCF file and annotated by ANNOVAR [19]. This VCF file including all mutational records and was used in the mutational signature analysis.

### Deciphering mutational signatures

The mutation signature was examined according to the matrix of 96 types of substitutions, including six substitution classes (C > A, C > T, C > G, T > C, T > A, T > G) along with substitutions in the context of right and left flanking bases. The non-negative matrix factorization (NMF) algorithm was applied to decompose the major k mutation signatures and their contributions [20]. k was selected based on the cophenetic correlations and the residual sum of squares [21]. The COSMIC Mutational Signatures database was applied as a reference for comparison and interpretation.

### Preparation of scRNA sequencing and T cell receptor sequencing libraries

Cell Ranger Single-Cell Software Suite was used to process the scRNA-seq data with default parameters and aligned to the GRCh38 reference genome. We removed those genes whose expressions were identified in < 0.1% of cells and filtered out cells that had mitochondrial RNA content > 20% or gene counts < 500 with Seurat package [22]. Genes which had highly variable expression were chosen according to the average expression and dispersion level thresholds by FindVariableGenes with default settings. The normalized expression for each gene were then regressed linearly against the total UMI counts using the ScaleData function and carried out the principal component analysis with RunPCA. We conducted graph-based Louvain clustering on 20 principal components with FindClusters. The marker genes for clusters were determined with Wilcoxon test implemented in FindAllMarkers function. The cells clusters were manually annotated according to these marker genes. The expression of genes and clustering results were plotted on a UMAP using RunUMAP [23].

### Inference of the unsupervised trajectory

After extracted the selected population from raw scRNA-seq data, Monocle [24], an algorithm using marker genes obtained from Seurat FindallMaker function, was employed to identify the differential states. With default settings, we constructed a spanning tree with DDRTree algorithm for cell ordering and dimension reduction.

### Gene set variation analysis (GSVA)

The pathway activities were estimated with the GSVA algorithm, which evaluate the pathway enrichment score by comparing the given gene expression matrix and the marker gene dataset from molecular signature database (MSigDB) [25]. The different pathways and *P* values were obtained from limma package.

### Statistical analysis

Kruskal-Wallis test (within multiple groups), Wilcoxon test (within two groups), and Fisher exact test were applied to analyze the comparisons among various categorical variables depend on the context. Survival curves were obtained from Kaplan-Meier plot and the log-rank test was used to evaluate the significance of differences. Hazard ratio and its 95% CI were calculated by Cox proportional hazards model. Two-sided *p* < 0.05 was considered statistically significant.

The statistical analysis was performed in R (4.3.0) with the help of packages of survival (v3.2), survminer (v0.4.9), meta (v4.9), Rtsne (v0.15), NMF (v0.23.0), mutSignatures (v2.1.1), dplyr (v1.0.6), forest plot (v3.1.3), plyr (v1.1.2), maftools (v2.16.0), clusterProfiler(v4.8.3), tidyverse (v2.0.0), rshape2(v1.4.4), ggsci (v3.0.0), scRepertoire (v1.0.2), copyKat (v1.0.8), and ggplot2 (v3.3.5).

## Results

### The sex dichotomy in the efficacy of immunotherapy

After carefully screening and selection, four trials were included for the final analysis based on our search strategy. The main characteristics of these studies were summarized in Suppl. Table 1. All four trials were international, multi-center, phase 3 RCTs. Due to the success of these eligible studies [3], FDA approved their applications in clinical practice in 2019 (KEYNOTE-181) [26], 2020 (ATTRACTION-3) [27], 2021 (KEYNOTE-590) [28], and 2022 (CHECKMATE-648) [29]. OS was the primary endpoint for all trials. The method qualities of these RCTs were generally good as evaluated by Jadad scores, the main issue affecting quality was lack of blinding since only KEYNOTE-590 was double-blind [28].

**Fig. 1:**
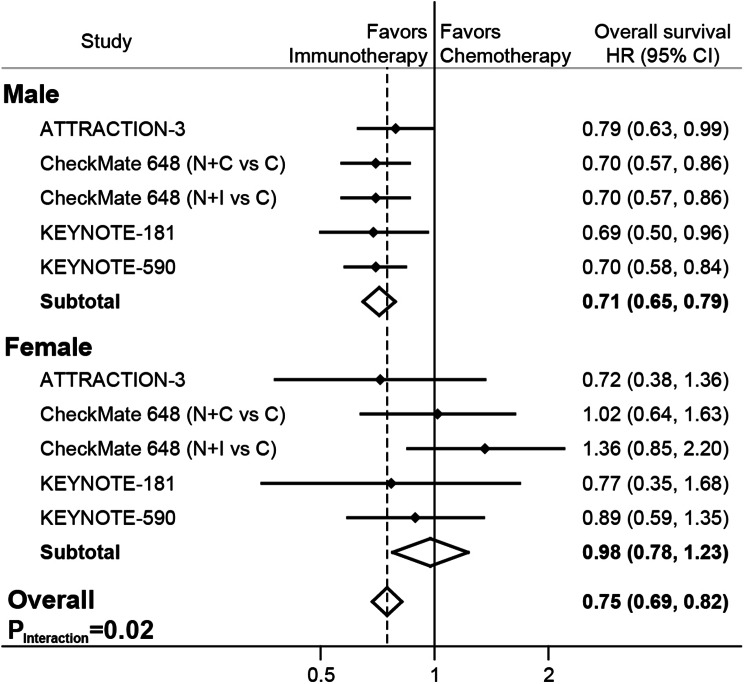
Comparison of the overall survival between immunotherapy and conventional chemotherapy in male, female, and overall population. C, chemotherapy; CI, confidence interval; HR, hazard ratio; I, ipilimumab; N, nivolumab

The analysis for OS included 2360 patients, most of them are men (*n* = 1977, 84%), and 1336 (57%) subjects were treated with ICIs. As expected, compared with conventional chemotherapy, ICIs decreased the risk of death by 25% (HR, 0.75; 95% CI, 0.69–0.82; *P* < 0.001; Fig. 1). However, further analysis revealed immunotherapy was associated with favorable outcomes only in men (HR, 0.71; 95% CI, 0.65–0.79; *P* < 0.001), but not in women (HR, 0.98; 95% CI, 0.78–1.23; *P* = 0.84). There was a significant difference in the efficacy of immunotherapy between male and female (*P*_interaction_=0.02). It should be noted that immunotherapy fails to show superior over chemotherapy in women in every single comparison. In contrast, in all the eligible trials, men can benefit from the application of ICIs, suggesting the dichotomy between male and female was robust and conclusive. No substantial heterogeneities were discovered in male (Q = 0.9; *I*^*2*^ = 0.0%; *P* = 0.92), female (Q = 3.3; *I*^*2*^ = 0.0%; *P* = 0.51), and overall population (Q = 10.2; *I*^*2*^ = 11.8%; *P* = 0.34).

### The genomic landscape of esophageal cancer

To explore the genomic mechanisms underlying the sex dichotomy, we selected, processed, and integrated WES/WGS information from 1425 EC patients in 13 datasets (male, *n* = 1100; female, *n* = 325). The key features of the eligible studies were presented in Suppl. Table 2. The potential impact of the heterogeneities in data collection, sequencing method, and analysis approach among various datasets were minimized with tremendous efforts in data verification. An overview of the pooled EC cohort was illustrated in Suppl. Figure 1. The frequencies of non-silent mutations remained relatively constant across various datasets (Suppl. Figure 1 A). The distributions of *t*-SNE clusters were characterized mainly by mutant genes, no obvious batch effects could be identified (Suppl. Figure 1B). Totally, we identified 134,049 non-silent mutations occurred in 17,572 genes. As shown in Suppl. Figure 1 C, the most common mutant genes were *TP53* (78%), *TTN* (34%), and *MUC16* (16%). Their frequencies in single datasets and overall dataset were similar to the pooled mutational frequencies.

**Fig. 2:**
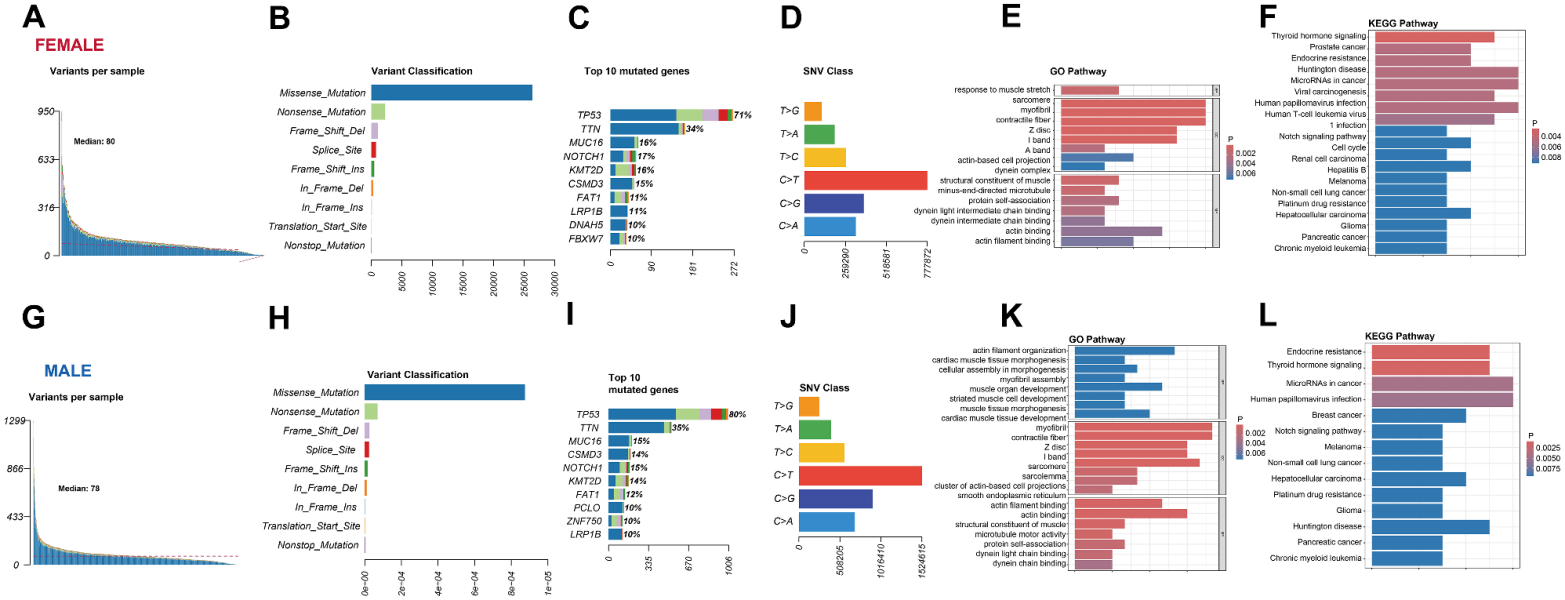
Genomic mutation landscape in female (upper panel) and male (lower panel) EC patients. The non-silent mutation burden (**A** and **G**), mutation subtype (**B** and **H**), top 10 most common mutant genes (**C** and **I**), SNV class (**D** and **J**), GO pathways (**E** and **K**), and KEGG pathways (**F** and **L**) in male and female EC patients. SNV, single nucleotide variant; GO, gene ontology; KEGG, Kyoto encyclopedia of genes and genomes

In female, 91.08% (*n* = 296) tumors harbored gene non-silent mutations, and the median numbers in every patient was 80 (interquartile range, 48–115; Fig. 2A). 83.70% of the mutations were missense mutation (Fig. 2B). The most common mutant genes were *TP53* (71%), *TTN* (34%), and *MUC16* (16%) (Fig. 2C), and the most common SNV class was C > T (Fig. 2D). We further examined the gene network affected by the most common 50 mutant genes in female by conducting GO analysis (Fig. 2E) and KEGG analysis (Fig. 2F). In male, non-silent mutations were discovered in 94.89% of the samples (*n* = 1040). Medially, 78 non-silent mutations were identified in every patient (interquartile range, 50–108; Fig. 2G). Missense mutations accounted for 82.25% (Fig. 2H). The highest mutant frequencies were found in *TP53* (80%), *TTN* (35%), and *MUC16* (15%) (Fig. 2I), and the most common SNV class was C > T (Fig. 2J). Similarly, we also investigated the gene network affected by the most common 50 mutant genes in men (Fig. 2K and L).

For genes whose mutant frequencies over 5%, only 8 genes showed significantly different between men and women (*P* < 0.05). The mutation of seven genes, namely *DMD, FBWX7, ZNF750, OBSCN, MUC4, FAT2*, and *DNAH11*, were enriched in female, while more *TP53* mutations were discovered in male. The gene network affected by these 8 mutant genes were illustrated in Suppl. Figure 2.

### The sex dichotomy of mutation signatures and reactome pathways

**Fig. 3:**
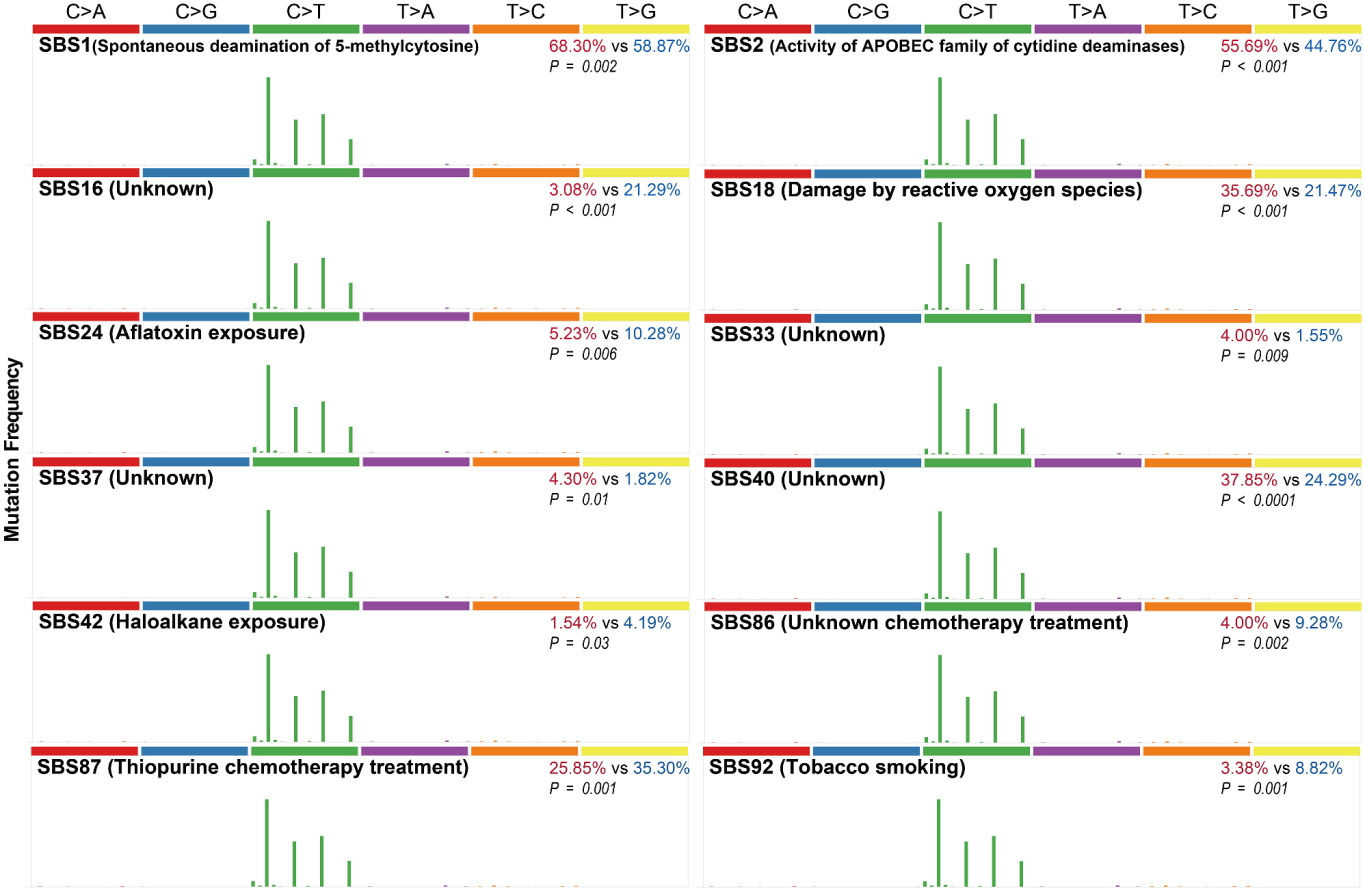
The frequencies of 12 COSMIC reference signatures were significantly different between male and female in EC. Bold black, SBS signature and its known etiologies. Red number, SBS frequency in female patients. Blue number, SBS frequency in male patients. *P* valued, the difference of SBS frequencies between male and female

Previous study of mutational processes revealed that both endogenous processes and exogenous exposures resulted in distinctive patterns of mutations, known as mutational signatures [30]. Here, we conducted non-negative matrix factorization analysis of mutational signatures with deconstructSigs [31], then the extracted mutation patterns were compared with Catalogue of Somatic Mutations in Cancer (COSMIC) reference signatures to estimate the mutation burden for each COSMIC signature. Totally, the frequencies of 78 mutational signatures (Suppl. Figure 3) were compared between male and female patients. As shown in Fig. 3, the frequencies of 12 signatures were statistically different between sexes. Among them, the frequencies of SBS1 (known etiology, spontaneous deamination of 5-methylcytosine), SBS2 (activity of APOBEC family of cytidine deaminases), SBS18 (damage by reactive oxygen species), SBS33 (unknown), SBS37 (unknown), and SBS40 (unknown) were increased significantly in women. However, SBS16 (unknown), SBS24 (Aflatoxin exposure), SBS42 (haloalkane exposure), SBS86 (unknown chemotherapy treatment), SBS87 (Thiopurine chemotherapy treatment), and SBS92 (tobacco smoking) were more common in men. Interestingly, C > T were enriched in all these SBS signatures.

**Fig. 4:**
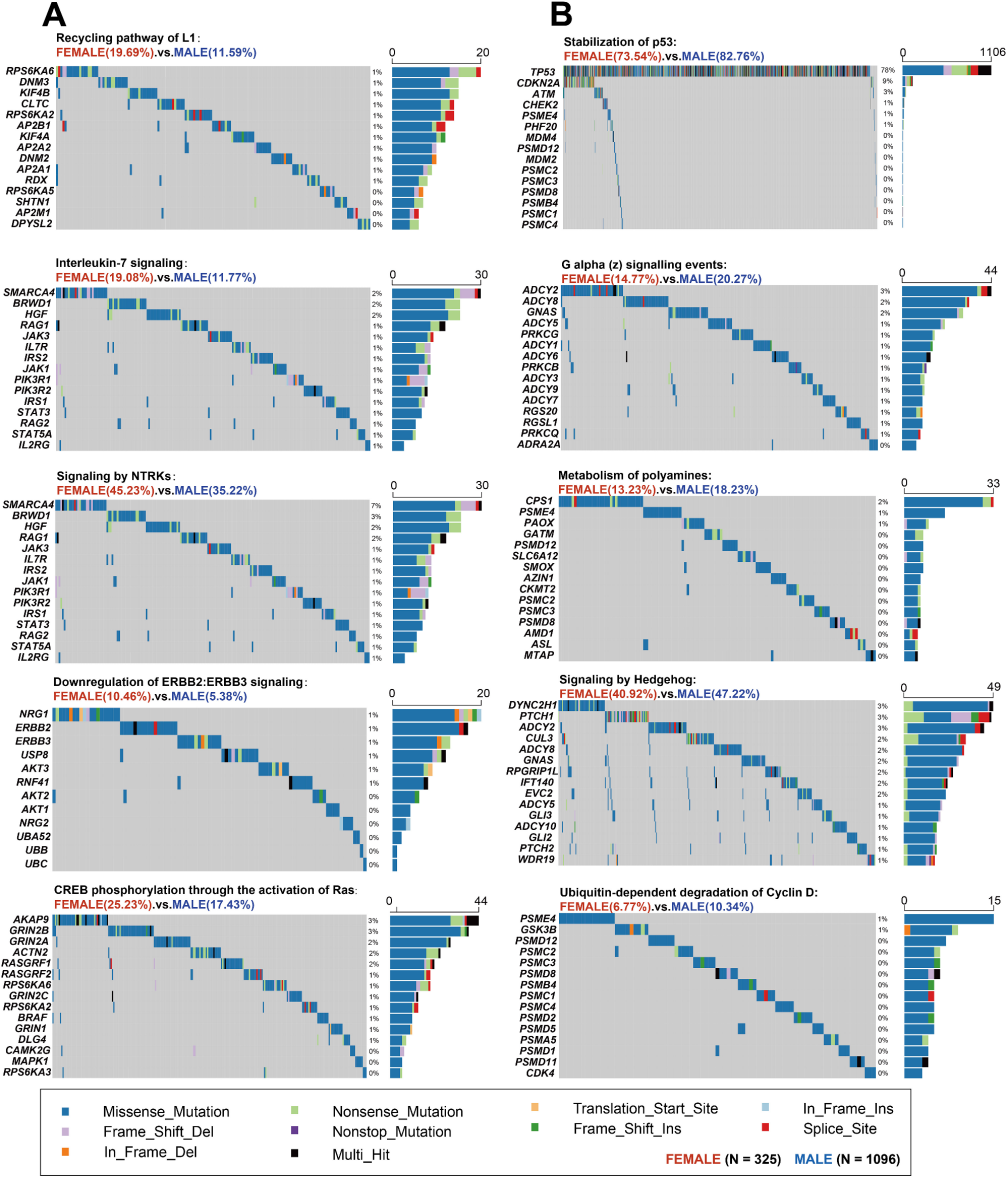
Ten typical altered reactome pathways occurred in EC patients. (**A**) Five pathways whose altered frequencies were significant higher in female. (**B**) Five pathways whose altered frequencies were significant higher in male

The reactome pathway knowledgebase, summarizing the molecular details of signal transduction, DNA replication, and other cellular processes, was a useful tool for discovering the functional associations from somatic mutation profiles in cancer [32]. With this knowledgebase, we investigated 2022 reactome pathways in 1421 eligible patients. As illustrated in Suppl. Figure 4, the most common altered reactome pathways occurred in EC were signal transduction (98.00%), immune system pathway (97.04%), and metabolism of proteins (97.04%). Most of the identified pathway alterations were missense gene mutations (85.90%). Moreover, we examined the alteration of 14 druggable genes, the biomarkers for potential targeted therapy. 14.39% (*n* = 205) patients harbored these druggable genes in EC, and the most common gene were *BRCA2* (3%), *BRCA1* (2%), *ROS1* (2%), *ALK* (2%), and *EGFR* (2%). Among these pathways, compared with female, the frequencies of 84 reactome pathways increased and 47 pathways decreased significantly in male (*P* < 0.05). In Fig. 4, we showed 5 typical pathways whose altered frequencies were significant higher in female (Fig. 4A), and 5 pathways that were higher in male (Fig. 4B). The full list of 131 altered reactome pathways was illustrated in Suppl. Table 3.

### Development and validation of a novel sex-related signature (SRS) to predict the efficacy of immunotherapy

Although the pooled analysis revealed that 8 mutations, 12 SBS, and 131 pathways were significantly different between male and female, none of these features demonstrated comparatively dispersive associations with the efficacy of immunotherapy in MSK cohort with 60 EC patients [33], suggesting single feature was insufficient to impact the whole landscape of anti-cancer immune response. Accordingly, we constructed a risk model to develop a comprehensive molecular signature that can predict the efficacy of immunotherapy.

A multivariable Cox regression analysis of the above candidate features was conducted for the OS in the MSK cohort [33], and 21 potential markers related to the efficacy of immunotherapy emerged. After careful evaluation, 6 features including 1 gene mutation (*FBWX7*) and 5 reactome pathways (signaling by NTRKs, regulation of RAS by GAPs, TP53 regulates transcription of DNA repair genes, DNA double-strand break repair, and FBXW7 mutants and NOTCH1 in cancer) were selected to construct as a risk model defined as SRS. The details of these 5 pathways and their frequencies in male and female were illustrated in Suppl. Figure 5. This model was calculated for every patient with the following formula derived from the alteration status (0 or 1) of the selected six features weighted by their regression coefficient:

SRS score = (0.81×TP53 regulates transcription of DNA repair genes) – (1.14×signaling by NTRKs) – (0.42×regulation of RAS by GAPs) – (1.90×FBXW7) – (0.38×DNA double-strand break repair) + (2.27×FBXW7 mutants and NOTCH1 in cancer).

**Fig. 5:**
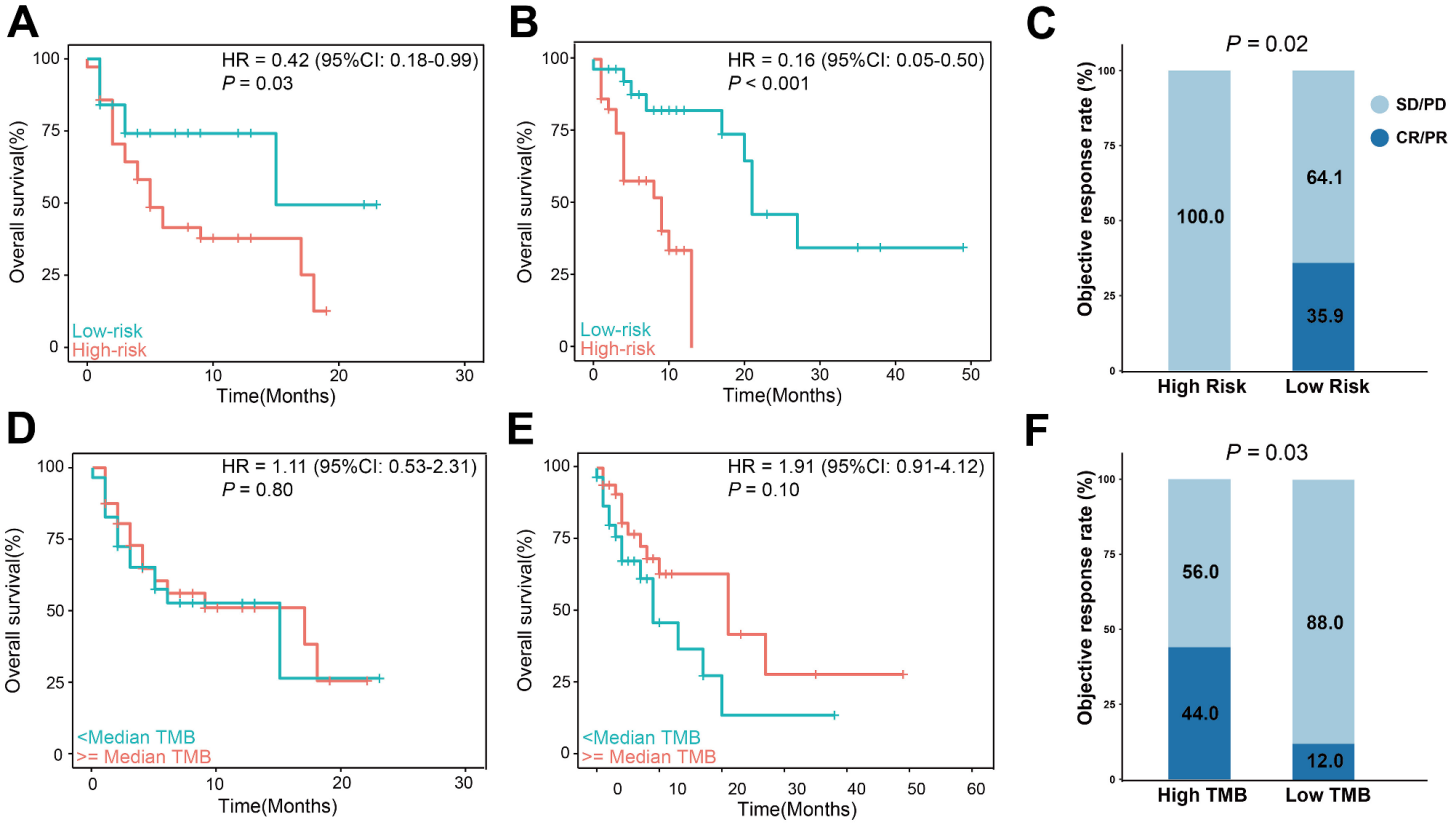
Development and validation of a novel sex-related signature (SRS) to predict the efficacy of immunotherapy. (**A** and **D**) Kaplan-Meier survival analysis in subgroups stratified by SRS (A) or TMB (D) in the training cohort with 60 EC patients. (**B** and **E**) Kaplan-Meier survival analysis in subgroups stratified by SRS (B) or TMB (E) in the validation cohort with 58 GC patients. (**C** and **F**) Comparison of objective response rates in subgroups stratified by SRS (C) or TMB (F) in another validation cohort with 55 GC patients. GC, gastric cancer; TMB, tumor mutation burden; CI, confidence interval; HR, hazard ratio; CR, complete response; PR, partial response; SD, stable disease; PD, progressive disease

Based on SRS score and OS, X-tile were applied to determine the optimal cutoff value and categorized patients into high-risk ( > = 1.31) and low-risk (< 1.31) subgroups. With this SRS model, 25 (41.7%) EC patients with low-risk score showed favorable outcomes compared with 35 patients (58.3%) with high-risk score (HR, 0.42; 95% CI, 0.18–0.99; *P* = 0.03) (Fig. 5A). To prove the generalization of SRS in predicting the efficacy of immunotherapy, we further evaluated the performance of this model in two cohorts enrolled patients with gastric cancer [33, 34]. As expected, low-risk score (*n* = 29, 50.0%) was associated with longer OS compared with high-risk score (*n* = 29, 50.0%; HR, 0.16; 95% CI, 0.05–0.50; *P* < 0.001) (Fig. 5B). In another cohort enrolled 55 patients, patients in low-score subgroup (*n* = 39) achieved higher objective response rate (ORR; 35.9% vs. 0.0%; *P* = 0.02; Fig. 5C) compared patients with high-score (*n* = 11). Tumor mutation burden (TMB) was an FDA-approved biomarker for immunotherapy [3]. As shown in Fig. 5D, E and F, the performances of TMB were not so powerful as our signature in predicting the efficacies of immunotherapy in all three cohorts.

### The sex dichotomy of tumor antigens in EC

Due to the differences in XY chromosomes, hormone levels, and genomics, the tumor antigens could induce different immune responses in male and female [35]. With the latest IMGT/HLA database as a reference, here we determined human leukocyte antigen (HLA) alleles with 6-digit precision using HLA-HD [36] in 183 patients with EC. The frequencies of all subtypes of HLA-I antigens (including HLA-A, HLA-B, and HLA-C) were similar between male and female. However, six subtypes of HLA-II antigens were enriched in women. The identified antigens and their prevalence were HLA-DMB*01:03:01 (female vs. male, 57.9% vs. 38.6%; *P* = 0.03), HLA-DOB*01:04:01 (21.1% vs. 7.6%; *P* = 0.02), HLA-DQB1*05:02:01 (26.3% vs. 12.4%; *P* = 0.04), HLA-DQB1*06:09:01 (10.5% vs. 1.4%; *P* = 0.02), HLA-DRB1*13:02:01 (10.5% vs. 2.1%; *P* = 0.03), and HLA-DRB1*14:54:01 (13.2% vs. 2.8%; *P* = 0.02).

### Single-cell transcriptome atlas of esophageal cancer

**Fig. 6:**
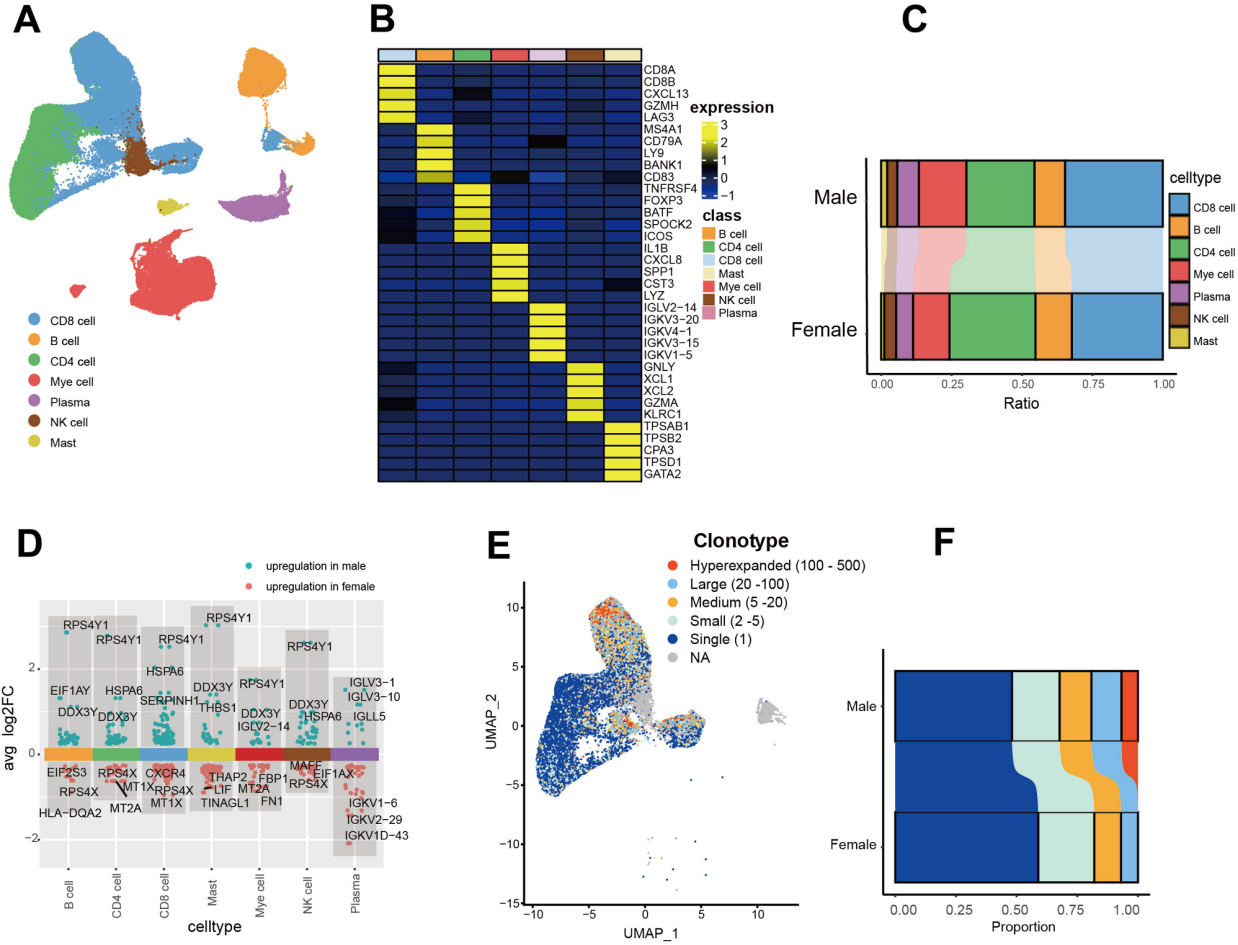
The single-cell transcriptome atlas of immunity in EC. Major subtypes of immune cells identified by uniform manifold approximation and projection (UMAP). Key marker genes for the classification of seven cell populations. Composition of different subtypes of immune cells in male and female EC patients. The differential expressed genes in male and female. The association between TCR clonotypes and immune cell populations. The proportions of different TCR clonotypes in male and female. TCR, T cell receptor

The tumor immune micro-environment played important roles in tumor growth, metastasis, and immunotherapy response [37]. To explore the cell populations within EC, we conducted scRNA-seq and T cell receptor (TCR)-seq analysis on immune cells from 60 patients [38] (Male, *n* = 44; female, *n* = 16; Suppl Table 4). After quality controls, we removed the batch effects and integrated the single-cell information with Harmony [39]. Totally, the transcriptome of 105,145 immune cells (CD45^+^) were included. Clusters obtained from the uniform manifold approximation and projection (UMAP) was annotated using established marker genes. As shown in Fig. 6A, seven major cell populations were identified: CD8^+^ T cells (*n* = 35,814), CD4^+^ T cells (*n* = 27,224), NK cells (*n* = 3952), myeloid cells (*n* = 16,605), plasma cells (*n* = 7635), B cells (*n* = 11,997), and Mast cell (*n* = 1918). The heat-map of marker genes of all seven cell populations were presented in Fig. 6B and Suppl Table 4.

Previous studies revealed that, compared with men, women tended to accumulate gene mutations which could strongly affect the mutation presented by MHC-II, hence a higher proportion of CD4^+^ T cell was observed in female than in male [40]. Here, we systematically investigated the composition of EC (Fig. 6C) and also found that there was a higher infiltration of CD4^+^ T cells in female compare with male patients (31% vs. 23%, *P* = 0.05). This trend remained relatively consistent across various tissue types and tumor stages. Interestingly, the proportions of other major types of immune were similar between men and women. The differential expressed gene (DEGs) between men and women in all 7 subtypes of immune cells were also examined (Fig. 6D and Suppl. Table 4), many DEGs played key roles in the physiological function of lymphocytes. We then investigated the richness of TCR clonotype in immune cells with scRepertoire [41]. As shown in Fig. 6E, most of the single or small clonotypes were identified in CD4^+^ T cells, while hyperexpanded, large, and medium clonotypes were mainly discovered in CD8^+^ T cells. As presented in Fig. 6F, sex dichotomy was also observed in term of proportions of clonotypes. Men harbored more hyperexpanded, large, and medium clonotypes, while single and small clonotypes were enriched in women.

### Exhausted CD8^+^ T cells were highly infiltrated in male patients with EC

**Fig. 7:**
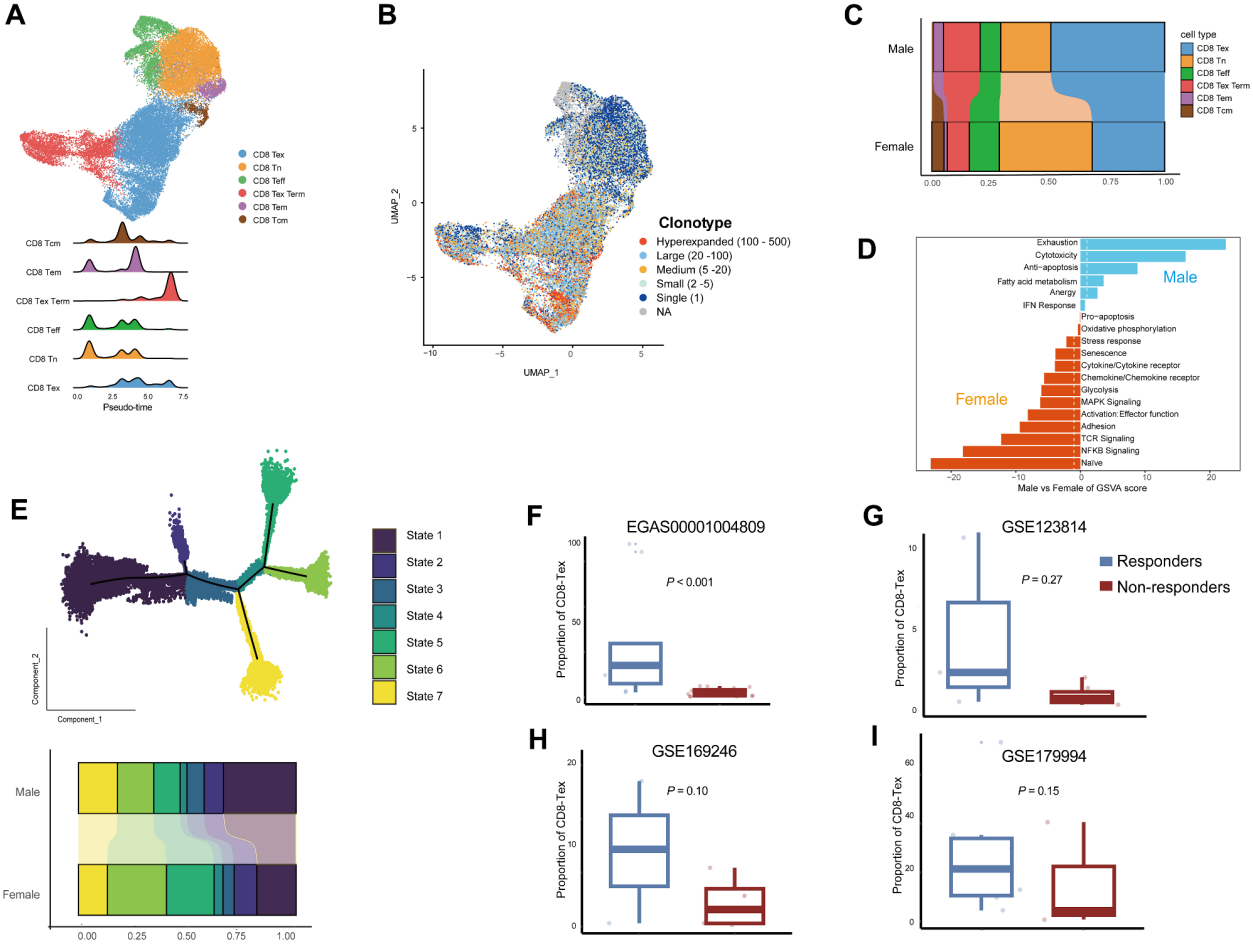
The key characteristics of CD8^+^ T cells in EC. (**A**) Major subtypes of identified CD8^+^ T cells. (**B**) The association between TCR clonotypes and CD8^+^ T cell populations. (**C**) Composition of different subtypes of CD8^+^ T cells in male and female. (**D**) Pathway activities estimated by GSVA in male and female. (**E**) The dynamic immune states of CD8^+^ T cells evaluated by Monocle and their proportions in male and female. (**F**-**I**) The proportion of exhausted CD8^+^ T cells in responders and non-responders in breast cancer (**F**), basal or squamous cell cancer (**G**), triple-negative breast cancer (**H**), and lung cancer (**I**)

Considering the central role of CD8^+^ T cells in cancer immunotherapy [35], next we investigated the characteristics of this specific cell population in male and female. As shown in Fig. 7A, six major subtypes of CD8^+^ T cells were identified in UMAP, namely exhausted CD8^+^ T cells (CD8-Tex, *n* = 14,170), naïve CD8^+^ T cells (CD8-Tn, *n* = 8300), effector CD8^+^ T cells (CD8-Teff, *n* = 3128), exhausted terminal CD8^+^ T cells (CD8-Tex-Term, *n* = 4520), Effector memory CD8^+^ T cells (CD8-Tem, *n* = 1173), and central memory CD8^+^ T cells (CD8-Tcm, *n* = 545). The marker genes used in cell classification were presented in Suppl Table 4. Further pseudo-time analysis confirmed the identities of these cell populations. Interestingly, TCR analysis revealed that single clonotypes were enriched in the naïve CD8^+^ T cells (Fig. 7B).

Next, we compared the abundances of different CD8 + subtype cells in male and female EC patients. As shown in Fig. 7C, compared with women, there were significant higher infiltrations of CD8-Tex (49% vs. 31%; *P* < 0.001), CD8-Tex-Term (16% vs. 9%; *P* < 0.001), and CD8-Tem (4% vs. 1%; *P* < 0.001) cells in men, while CD8-Tn (22% vs. 40%; *P* < 0.001) and CD8-Tcm (0.5% vs. 5%; *P* < 0.001) were less abundant. The proportion of CD8-Teff were similar between two subgroups. Totally, the exhausted CD8^+^ T cells accounted for 65% of all identified CD8^+^ T cells in men. We then conducted GSVA analysis on CD8^+^ T cells in male and female patients, respectively. As illustrated in Fig. 7D, exhaustion and cytotoxicity signature pathways were highly enriched in men, while naïve signaling pathway obtained the highest score in women. These results further confirmed the robust sex dichotomy in the compositions of CD8^+^ T cells. We also deciphered the dynamic immune state by inferring the state trajectories with Monocle [24]. As shown in Fig. 7E, seven states along the pseudo-developmental stages were identified in CD8^+^ T cells. More late-stage of CD8^+^ T cells were discovered in male, while early-stage of CD8^+^ T cells were enriched in female.

Based on these results, we hypothesized the proportion of CD8-Tex could be a powerful predictive biomarker in immunotherapy. Indeed, we evaluated the performance of this predictor in four independent cohorts, namely EGAS00001004809 (breast cancer; responders vs. non-responders, 9 vs. 18; Fig. 6F) [42], GSE123814 (basal or squamous cell cancer; responders vs. non-responders, 3 vs. 5; Fig. 6G) [43], GSE169246 (triple-negative breast cancer; responders vs. non-responders, 3 vs. 4; Fig. 6H) [44], and GSE179994 (lung cancer; responders vs. non-responders, 6 vs. 3; Fig. 6I) [45]. As expected, in all four cohorts, the proportion of CD8-Tex in responders were higher than those in non-responders. We also assessed the predictive value of naïve CD8^+^ T cells in these cohorts. As demonstrated in Suppl. Figure 6, the performance of CD8-Tn as a biomarker was found to be only marginally effective.

### Identification and characterization of other cell populations in male and female

Five subtypes of B cells were identified in EC (Suppl. Figure 7 A). The marker genes for classification were presented in Suppl. Figure 7B and Suppl Table 4. The proportions of these five subtypes of B cells showed no significant difference between female and male (Suppl. Figure 7 C). Similar analysis was also conducted on CD4^+^ T cells (Suppl. Figure 8), and macrophage and DC cells (Suppl. Figure 9). No substantial sex dichotomies were discovered in these cell populations. CopyKAT [46], a computational tool to separate normal cells from malignant cells, was applied to study the epithelial cells. The proportion of ten subtypes of epithelial cells were also similar between male and female (Suppl. Figure 10).

Interestingly, we discovered the abundance of one specific subgroup of NK cells were significantly increased in male patients (Suppl. Figure 11). The featured maker gene in these NK cells was *BAG3*, which could mediate the CD8^+^ T cell recruitment [47]. Further investigations were needed to disclose the underlying mechanisms between this specific cell population and the efficacy of cancer immunotherapy.

## Discussion

Enhancing the immune response lies in the center to the new wave of immunotherapies, and deciphering the differences in efficacy due to sex disparities is particularly interesting [5]. However, in EC, the underlying molecular basis of sex dimorphism and their impacts on the outcomes of immunotherapy is poorly understood. Here, for the first time, with 2360 EC patients from phase 3 RCTs, we revealed that only men could benefit from ICIs. In women, immunotherapy failed to show superior over chemotherapy. To explore the underlying mechanisms, we systematically characterized the genomic landscapes of EC with 1425 patients, and found the frequencies of 8 gene mutations, 12 mutation signatures, 131 reactome pathways, and 6 subtypes of HLA-II antigens were significantly different between male and female. Hence a novel sex-related signature based on was constructed, and this signature was more powerful than convention TMB as a biomarker to predict the efficacy of immunotherapy. Furthermore, we conducted scRNA-seq and TCR-seq analysis on 105,145 immune cells from 60 EC patients. Interestingly, exhausted CD8^+^ T cells were notably enriched in men (65%), while the most common subtype of CD8^+^ T cell occurred in female was naïve CD8^+^ T cells (40%). These results might explain the sex dichotomy in the efficacy of immunotherapy. Our study might have implications in treatment decision-making, design/interpretation of clinical trials, and personalization of immunotherapy.

Currently, it is still in controversial whether there are sex-biased efficacies in patients treated with ICIs [8]. Accumulating evidences suggest that sex demonstrates different impact on the outcome of immunotherapy in different tumor types. In melanoma, more male patients showed favorable survival outcomes [10–13], similar results were also observed in colorectal cancer [48]. While in lung cancer, immunotherapy can significantly improve the overall survival in women [9]. In EC, it is well-established the cases of male patients were significant more than female cases across various age, region, and tumor stage [1], suggesting the biological basis associated with sex disparities play a significant role in this particular malignancy. Here, we conducted a meta-analysis to specifically examined the sex disparities based on four phase 3 RCTs that led the application of immunotherapy in clinical practice granted by FDA, and found that female could not benefit from immunotherapy. Indeed, for women with EC, immunotherapy failed to show superior over conventional treatment in every single comparison. By contrast, the benefits of ICIs in male patients were significant in every eligible trials. The robust disparities in the efficacy of immunotherapy confirmed there was a sex dichotomy in tumor micro-environment of EC.

Although the adaptive and innate immunity in women has been reported to be more active than in men [49], the exact differences in tumor, especially in EC, between genders remained yet to be clarified. Here, with genomic information collected from approximately 1,500 EC patients, we first investigated the whole genomic landscapes and discovered there were no significant differences between male and female in term of non-silent mutation burdens, most common mutant genes, the composition of different subtypes of mutations. While we failed to discovered large sex bias in gene mutations, we speculated that it was possible some mutations might demonstrate differences in their potential to function as neoantigens when the underlying mutational processes were active at different times or were biased in their protein presentations [40]. These findings were consistent with previous reports revealing there was no difference in TMB between male and females in several types of tumors [6, 50]. Although TMB has been granted by FDA as a predictive biomarker for immunotherapy, it is an imperfect predictor with multiple technical limitations [51]. Meanwhile, single gene mutation was insufficient to change the whole tumor microenvironment and unlikely to be a comprehensive biomarker for immunotherapy. Currently, it was well-established that risk models with selected molecular characteristics can be better cover the shortages of existing biomarkers [35]. Here, we constructed a novel model based on six genomic features (one gene, five reactome pathways). Each feature could exert impact on the immune contexture or immune response. TP53 regulated transcription of DNA repair genes that led to the activation of innate and adaptive immune responses [52]. FBXW7 maintained the maturity and function of immune cells by regulating the ubiquitination-dependent degradation of substrate proteins [53]. Notch signaling though NOTCH1 was essential for the development of T cells [54]. The crosstalk between DNA damage repair and innate immunity was compelling for the immune functions [55]. RAS signaling pathway mediated the recruitment, differentiation, and activation of immune cells during the tumor cells to evading immune surveillance process [56]. Accordingly, our risk model had theoretical rationality and biological meaning. More importantly, this signature held great promise by its broad applicability giving its robustness and stability in predicting the outcomes of gastric cancer in two independent cohorts.

The success of immunotherapies to induce favorable outcomes in some patients relied heavily on T cell recognition of tumor antigens [35]. As ICIs had limited efficacy, tumor antigens have the potential to be examined for complementary treatments. It was reported that, due to the genetic and hormonal factors, female tended to harbor gene mutations that could strongly affect the mutation presented by MHC-II compared with male, hence a higher CD4^+^ T cell counts was observed in women [40, 49]. Consist with previous studies, we also discovered the strong effects in MHC-II based selection, but not in MHC-I antigens. Further single cell sequencing analysis was also in agreement with the fact that females had a higher proportion of CD4^+^ T cell.

The differentiation of CD8^+^ T cell is a tightly regulated process, context and duration of antigen can determine the trajectory of CD8^+^ T cell differentiation. The hallmark of the CD8^+^ T-cell response is exhaustion, a dysfunctional state due to the adaptation to long-term antigen exposure [57]. During exhausted process, persisting CD8^+^ T cells undergo a hierarchical loss of functions leading to a state of hypo-responsiveness [58]. Specifically, exhausted T cells enhance the expression of PD-1, which prompt the development of antibodies targeting PD-1/PD-L1 [59]. These ICIs can restore the function of CD8^+^ T cell, and a higher level of CD8^+^ T infiltration (“infiltrated type”) is reported to predict a better prognosis in many cancer types [60, 61]. The reasons behind this phenomenon may partially be due to the fact that PD-L1 gene is regulated by estrogens and X-linked mRNA [62]. Exhausted CD8^+^ T cells also express a series of other cell surface inhibitory molecules such as CD244, CTLA-4, LAG-3, CD160, and TIM-3 [63]. These co-expression of PD-L1 and other receptors pattern are functionally relevant since simultaneous blockade of multiple targets lead to the synergistic reversal of exhaustion. As shown in our clinical meta-analysis, the immunotherapy combination treatments were applied as first-line treatment. In EC, our analysis revealed that exhausted CD8^+^ T cells accounted for about two third of all the CD8^+^ T cell in male patients. This high proportion of exhausted CD8^+^ T cells could easily explain why immunotherapy was so effective in men. By contrast, the most common subtype of CD8^+^ T cell occurred in female was naïve CD8^+^ T cells (40%). Antibodies targeting PD-1 or PD-L1 hardly impact the physiological function of this specific kind of cells [35], hence women cannot benefit from immunotherapy, as we discovered in our meta-analysis. Further investigation on the dynamic immune state of CD8^+^ T cells also confirmed that, compared with female, more T cells identified in male were in their developmental late-stage. These results strongly suggested that the proportion of exhausted CD8^+^ T cells could be a powerful biomarker indicating the efficacies of immunotherapy. Indeed, we validated its predictive value in multiple malignancies, suggesting the generalization of this biomarker in predicting the efficacy of immunotherapy.

The molecular mechanisms of the sex disparity in CD8^+^ T cells are incompletely understood. However, it was revealed that, in animal tumor models, the frequency of stem cell–like CD8^+^ T cells decreased while the frequency of terminally exhausted CD8^+^ T cells were increased along with higher PD-1 expression [64]. Further investigation of publicly available integrated transcriptomic data demonstrated androgen receptor (AR) expression were enriched in CD8^+^ T cells compared to other T cell subtypes [65]. When AR-sufficient and AR-deficient CD8^+^ T male mice were compared, AR-deficiency decreased the proportion of exhausted cells and enhanced effector cytokine and Granzyme B production [64, 65]. Recently, Kwon et al. reported that intrinsic AR could directly regulate *Tcf7/*TCF1, a key transcriptional trans-activator participated in the fate decisions of CD8^+^ T cells [66]. It has been established that this TCF1^+^CD8^+^ exhausted T cell subtype possesses tumor-antigen specificity and exhibits the ability to maintain durable immunity, particularly when exposed to ICIs [67]. Meanwhile, it was also proposed that the inhibitory functional of CD8^+^T cell-intrinsic AR pathway was suppressed directly by *IFNG* in prostate cancer immunotherapy [68]. Both studies serve as valuable supplements to one another and offer potential directions for future research in AR’s critical role as a regulator of tumor immunity.

## Perspectives and significance

In esophageal cancer, our analysis based on bulk and scRNA-seq data demonstrated that there were robust genomic and cellular sex disparities. Furthermore, compared with conventional chemotherapy, only men could benefit from immunotherapy. These results may assist in treatment decision-making, design/interpretation of clinical trials, and personalization of immunotherapy.

### Electronic supplementary material

Below is the link to the electronic supplementary material.

Supplementary Material 1

Supplementary Material 2

Supplementary Material 3

Supplementary Material 4

Supplementary Material 5

Supplementary Material 6

Supplementary Material 7

Supplementary Material 8

Supplementary Material 9

Supplementary Material 10

Supplementary Material 11

Supplementary Material 12

Supplementary Material 13

Supplementary Material 14

Supplementary Material 15

## Data Availability

The datasets generated during and/or analyzed during the current study are available from the corresponding author upon reasonable request.
