# Supplementary figures and images for: Sex disparities revealed by single-cell and bulk sequencing and their impacts on the efficacy of immunotherapy in esophageal cancer
Source: Biol Sex Differ. 2024 Mar 15;15:22. doi: 10.1186/s13293-024-00598-z (PMC10941500; doi:10.1186/s13293-024-00598-z)

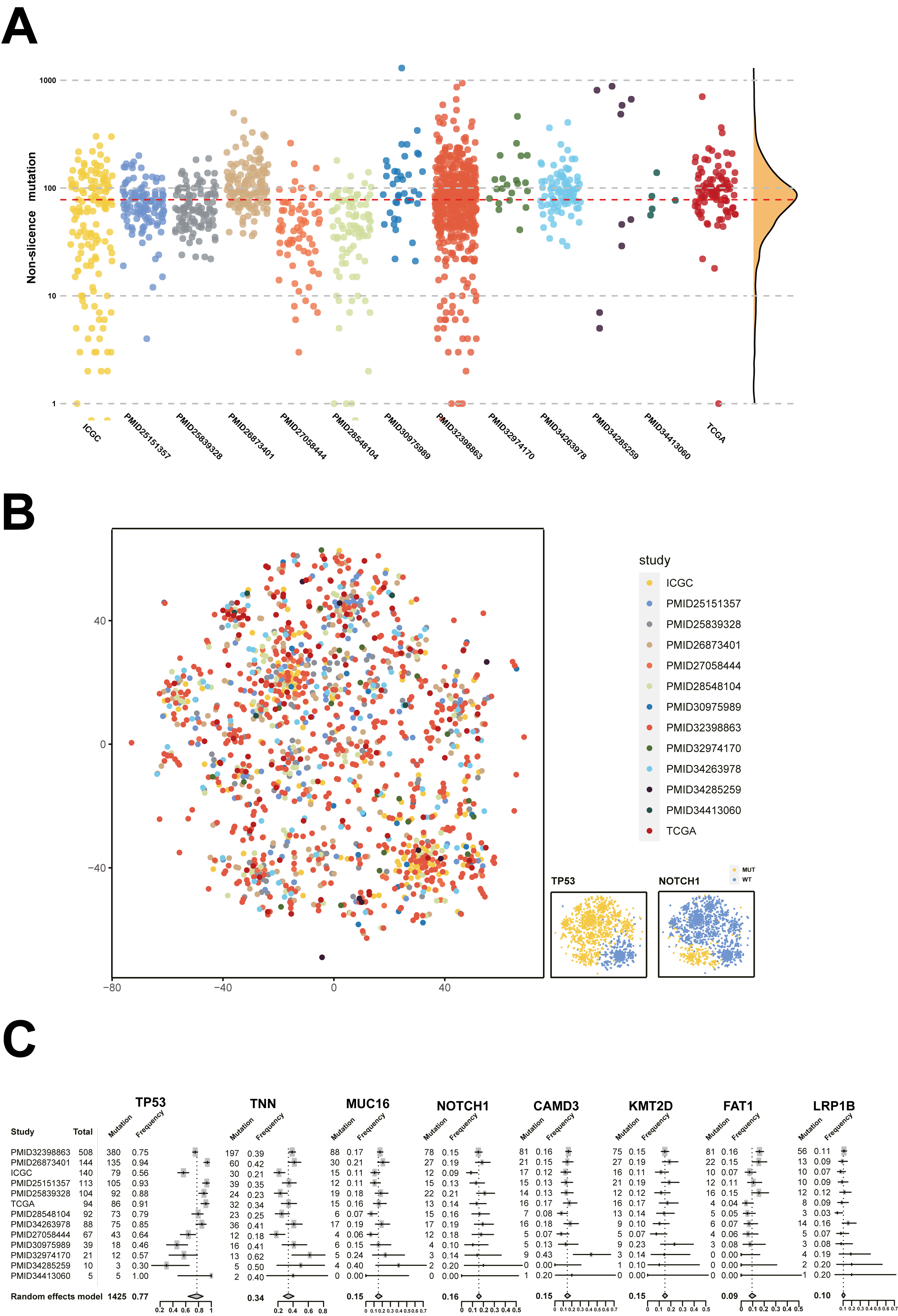

Supplement: Supplementary file 3 — Supplementary Material 3 [file 13293_2024_598_MOESM3_ESM.jpg]

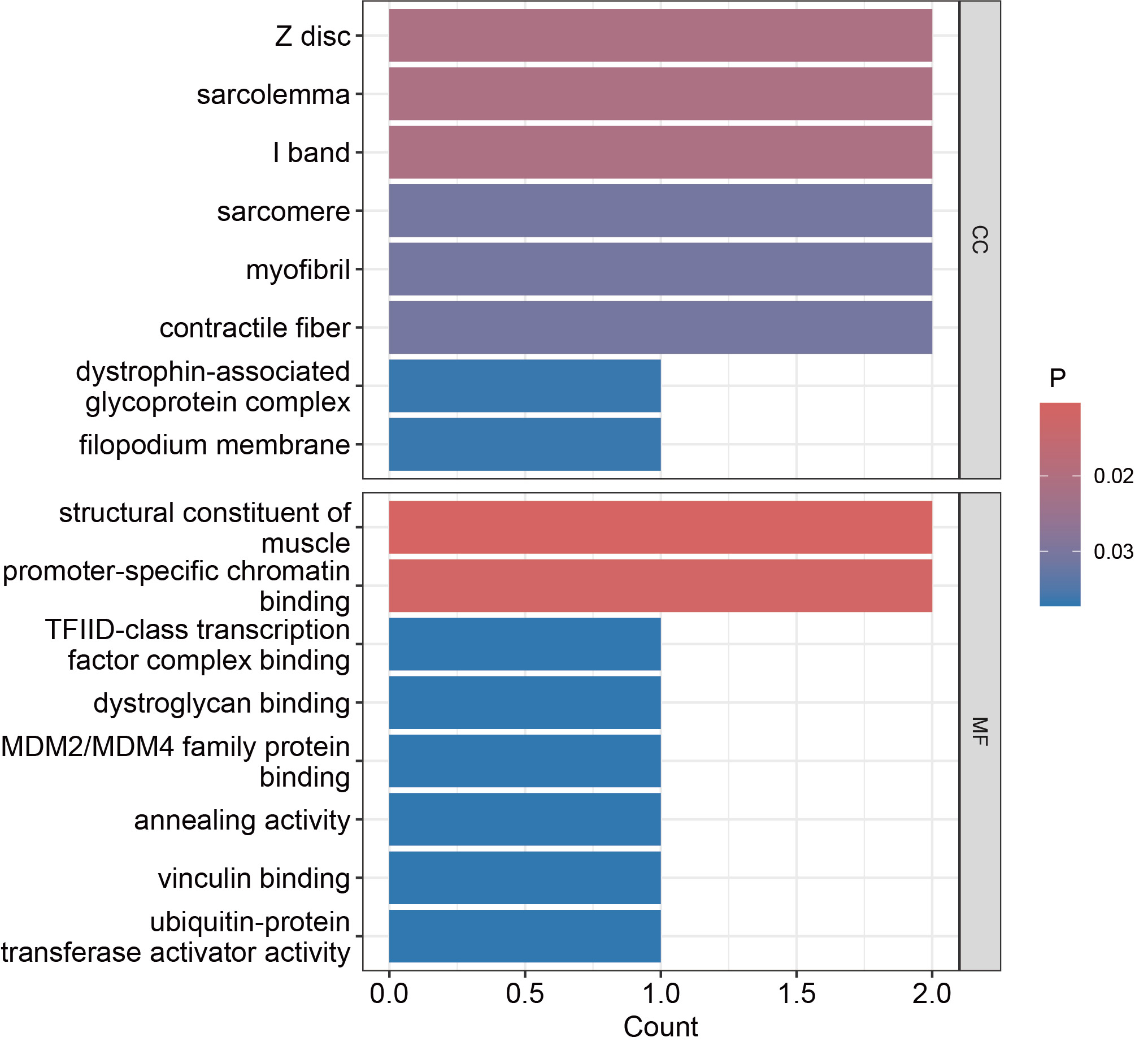

Supplement: Supplementary file 4 — Supplementary Material 4 [file 13293_2024_598_MOESM4_ESM.jpg]

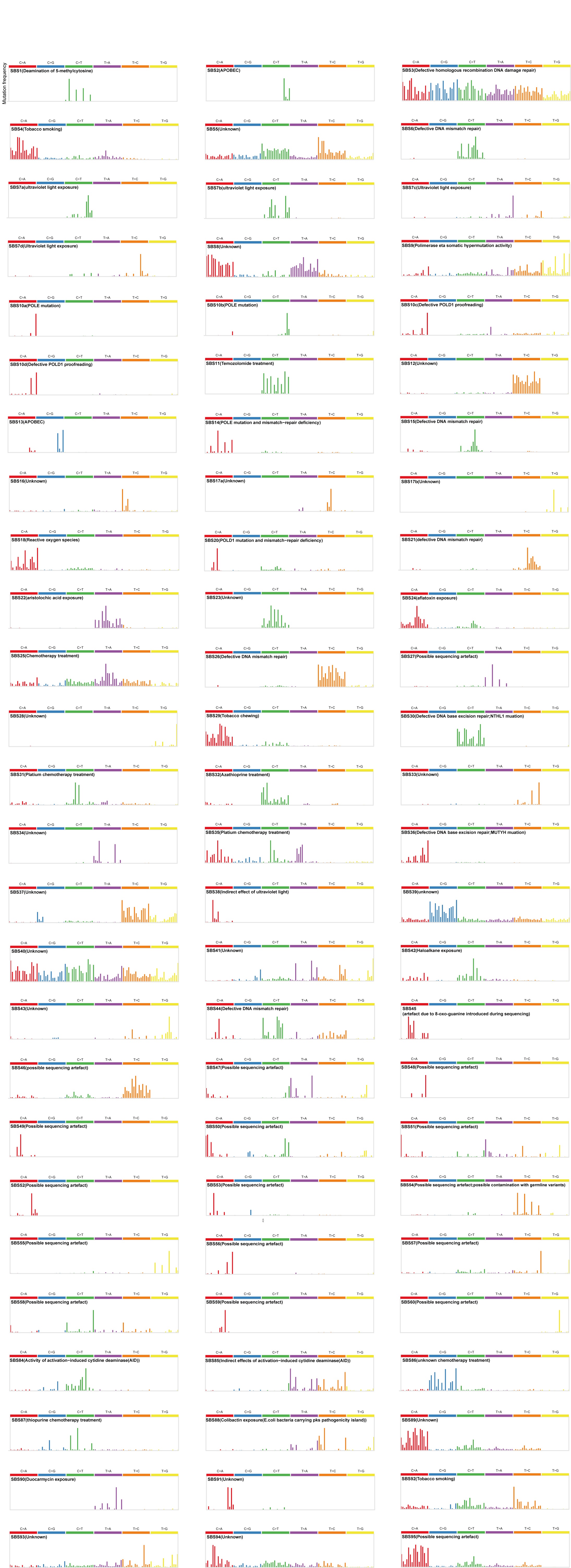

Supplement: Supplementary file 5 — Supplementary Material 5 [file 13293_2024_598_MOESM5_ESM.jpg]

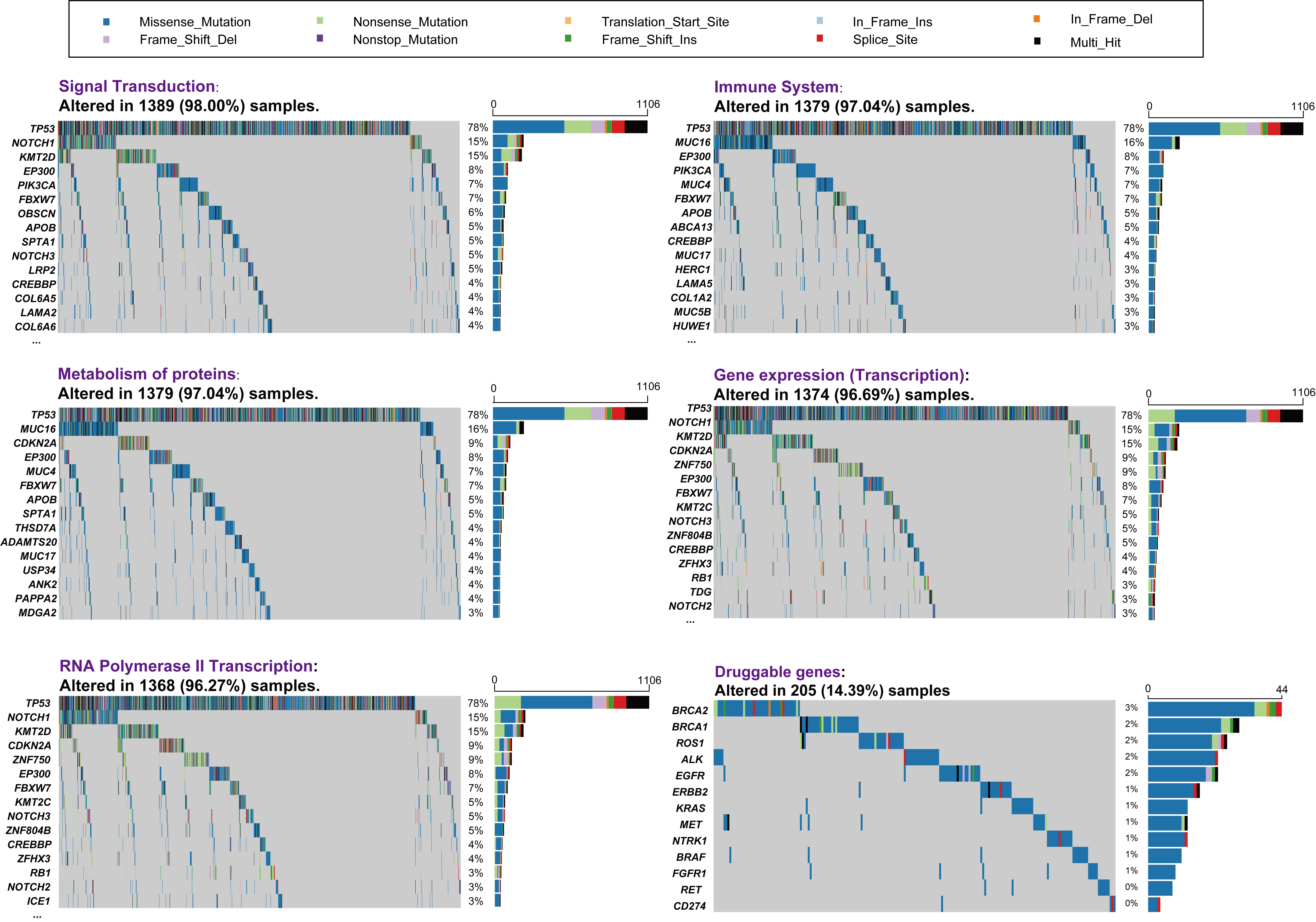

Supplement: Supplementary file 6 — Supplementary Material 6 [file 13293_2024_598_MOESM6_ESM.jpg]

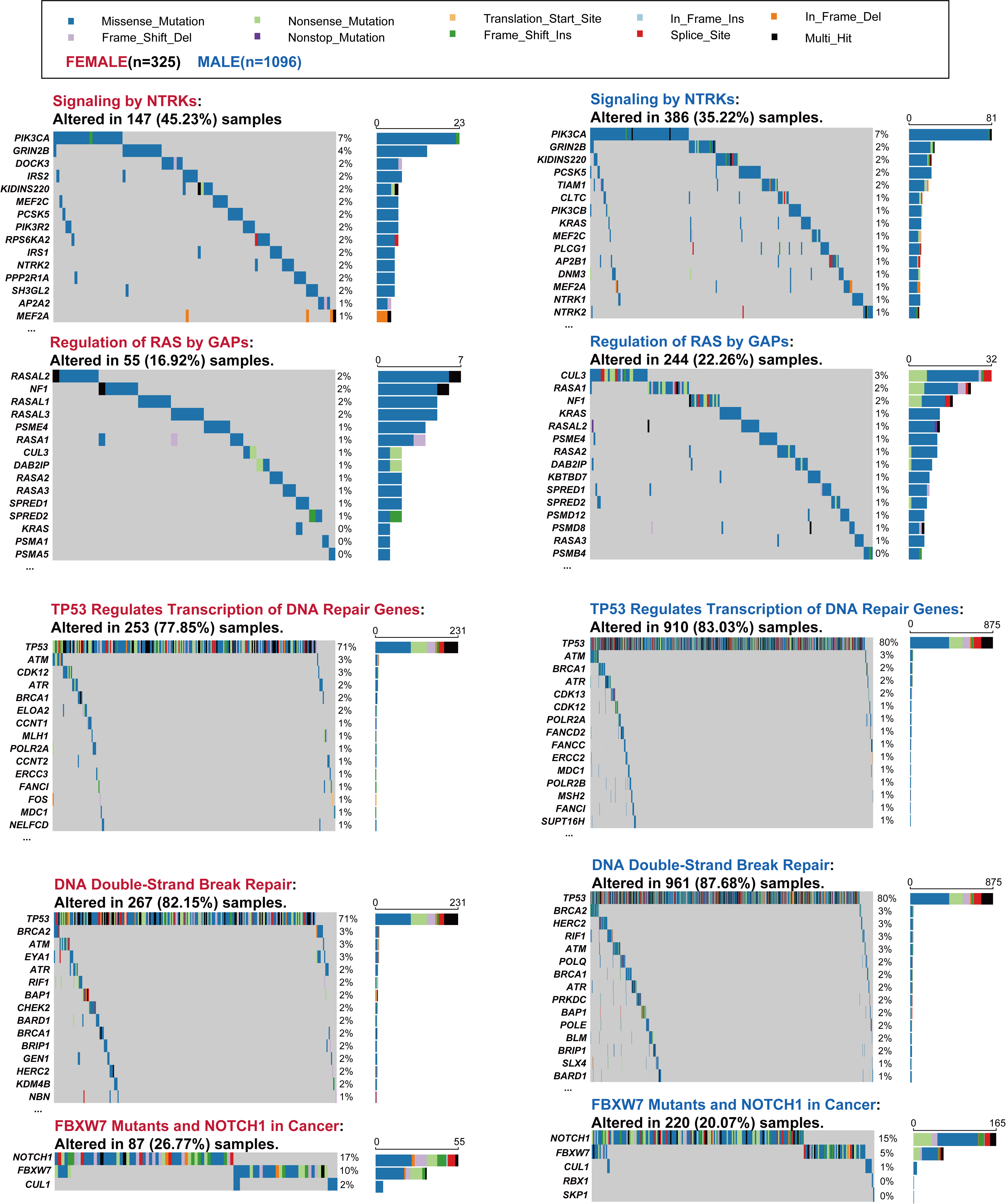

Supplement: Supplementary file 7 — Supplementary Material 7 [file 13293_2024_598_MOESM7_ESM.jpg]

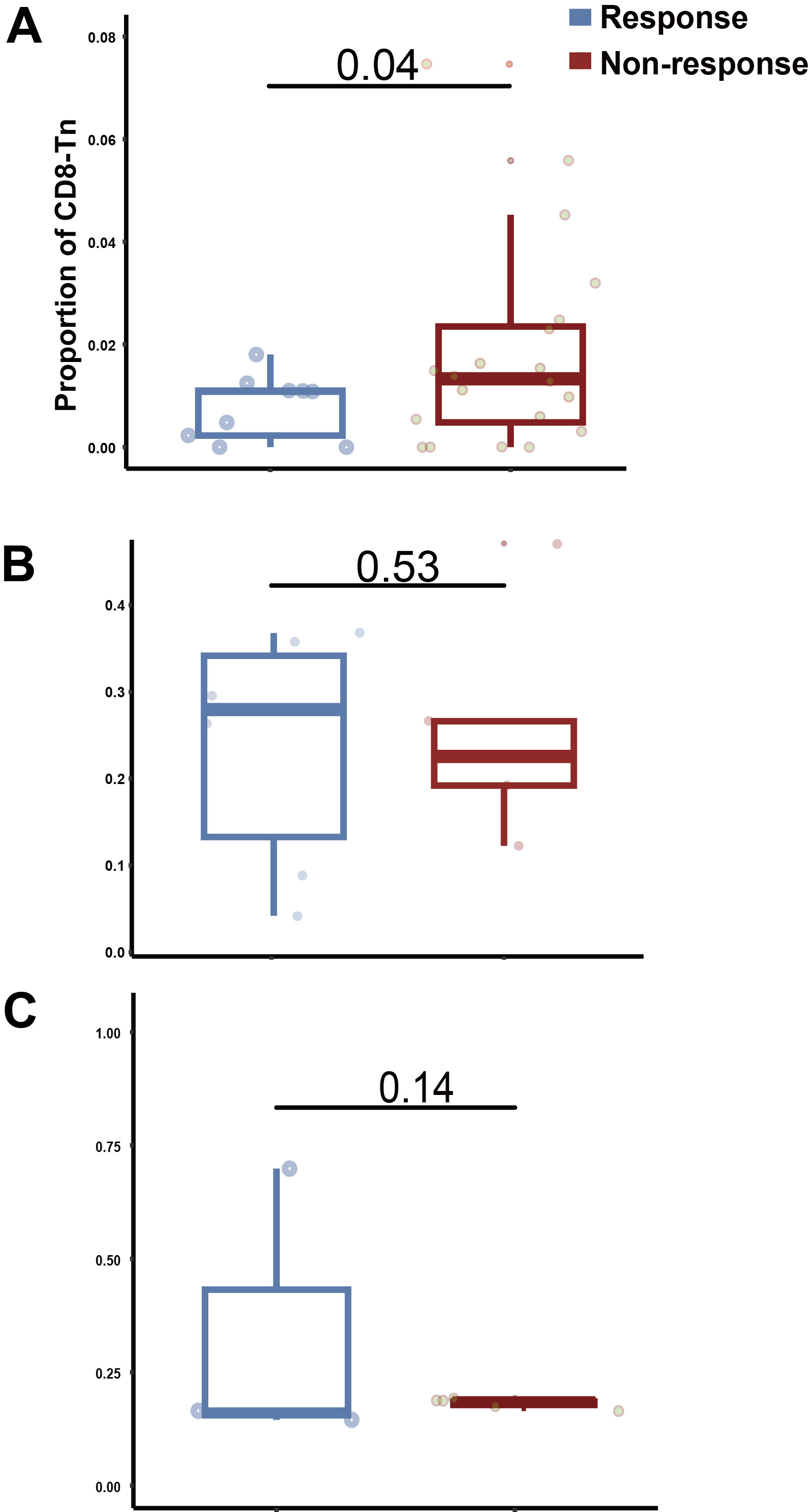

Supplement: Supplementary file 10 — Supplementary Material 10 [file 13293_2024_598_MOESM10_ESM.jpg]

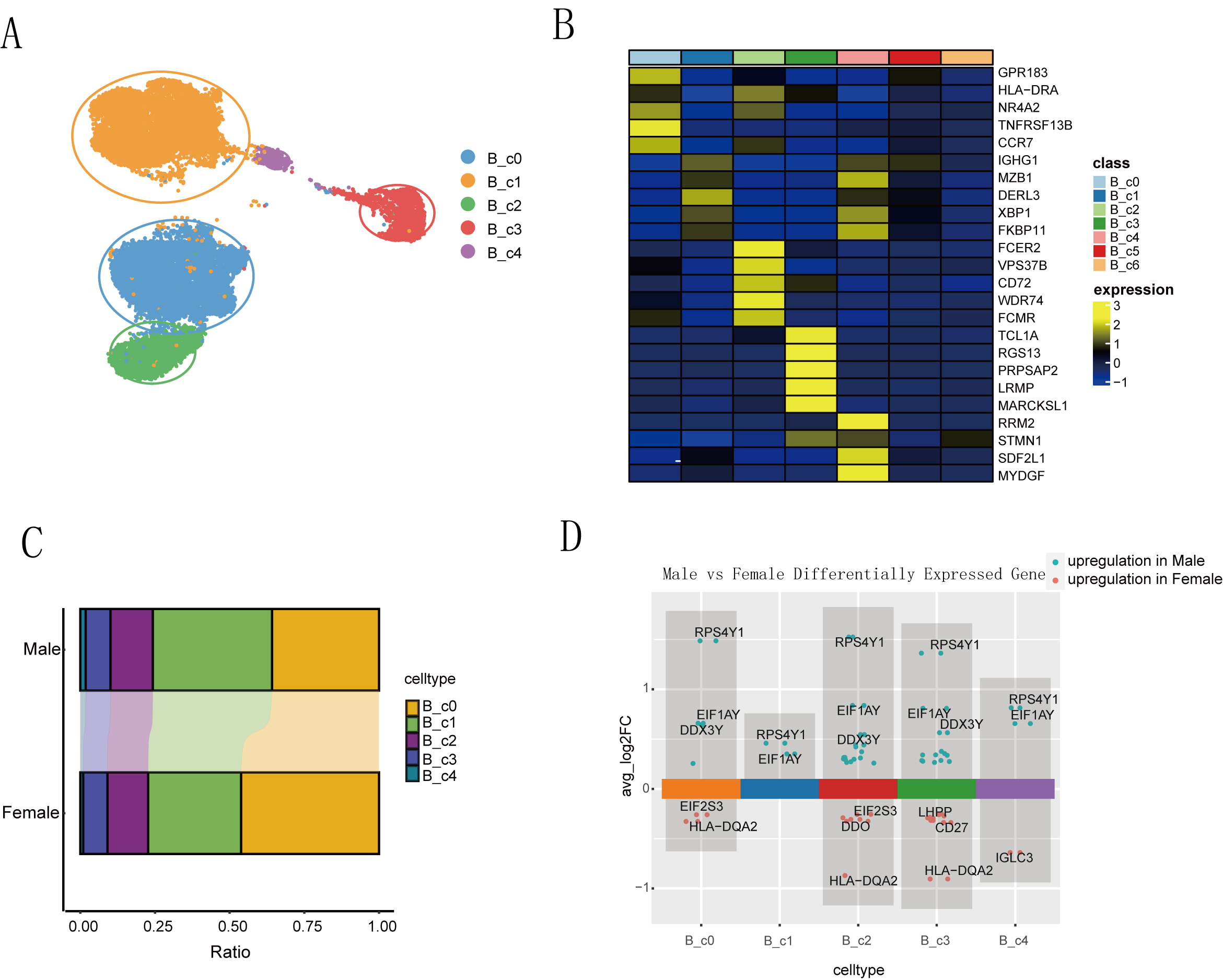

Supplement: Supplementary file 11 — Supplementary Material 11 [file 13293_2024_598_MOESM11_ESM.jpg]

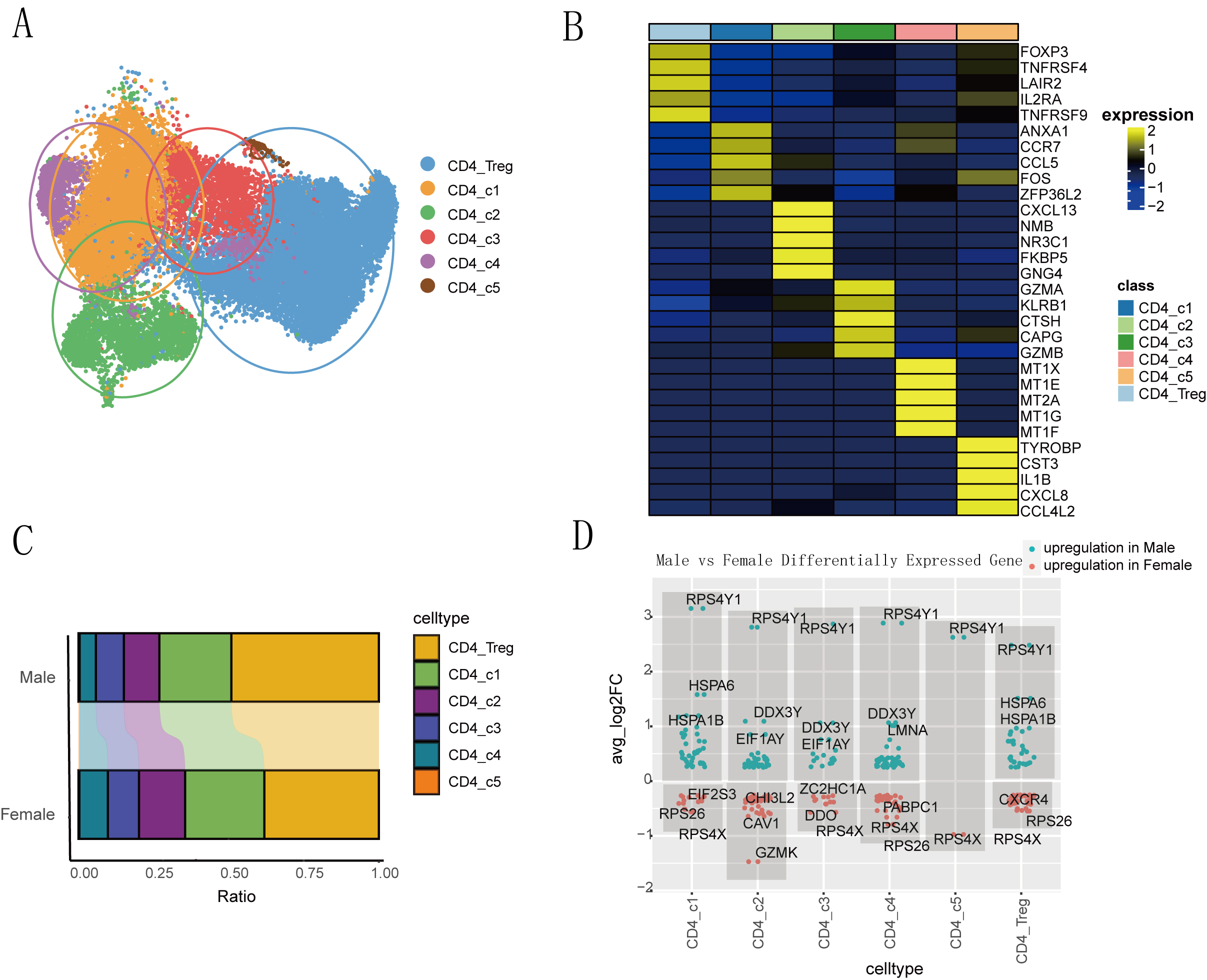

Supplement: Supplementary file 12 — Supplementary Material 12 [file 13293_2024_598_MOESM12_ESM.jpg]

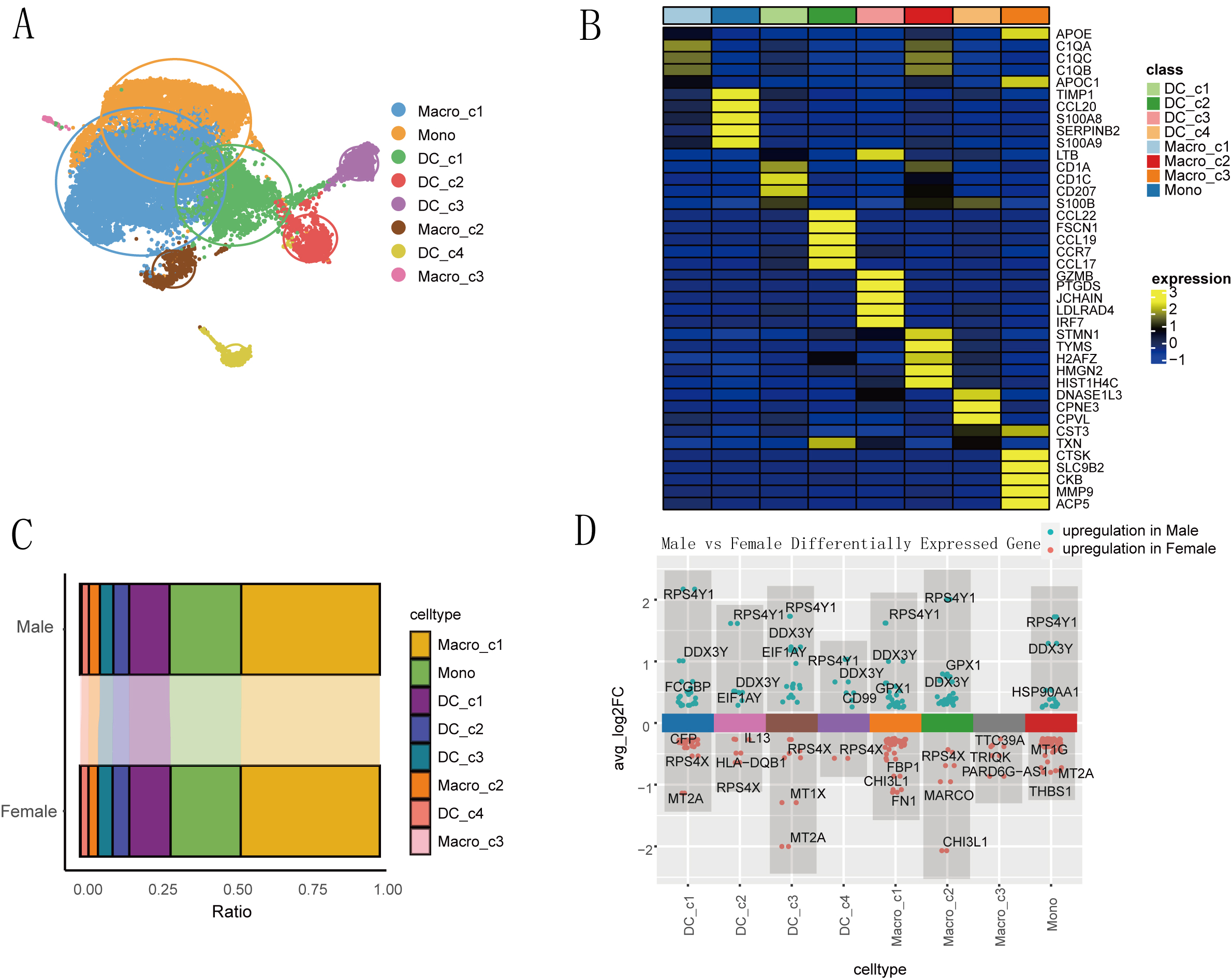

Supplement: Supplementary file 13 — Supplementary Material 13 [file 13293_2024_598_MOESM13_ESM.jpg]

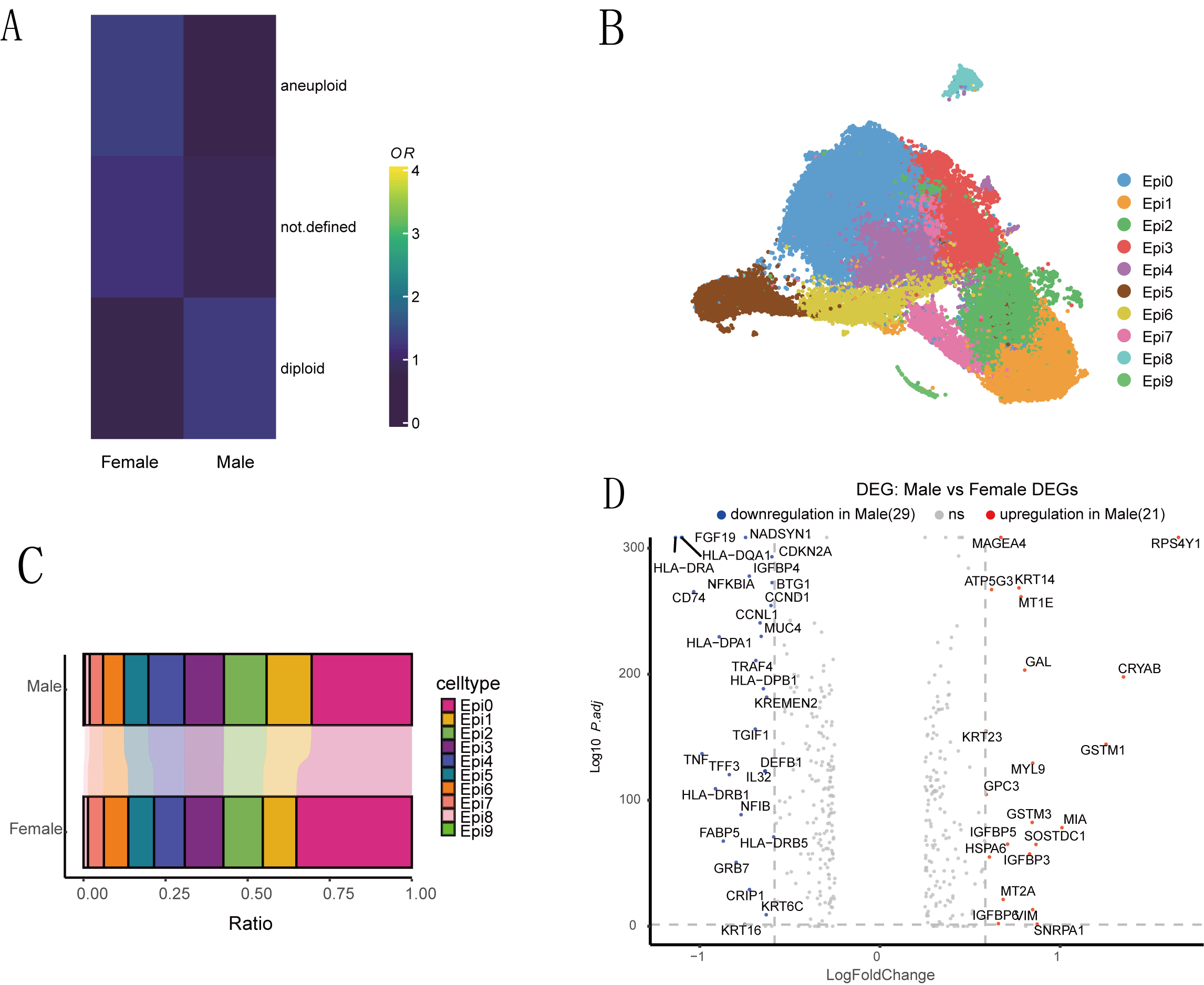

Supplement: Supplementary file 14 — Supplementary Material 14 [file 13293_2024_598_MOESM14_ESM.jpg]

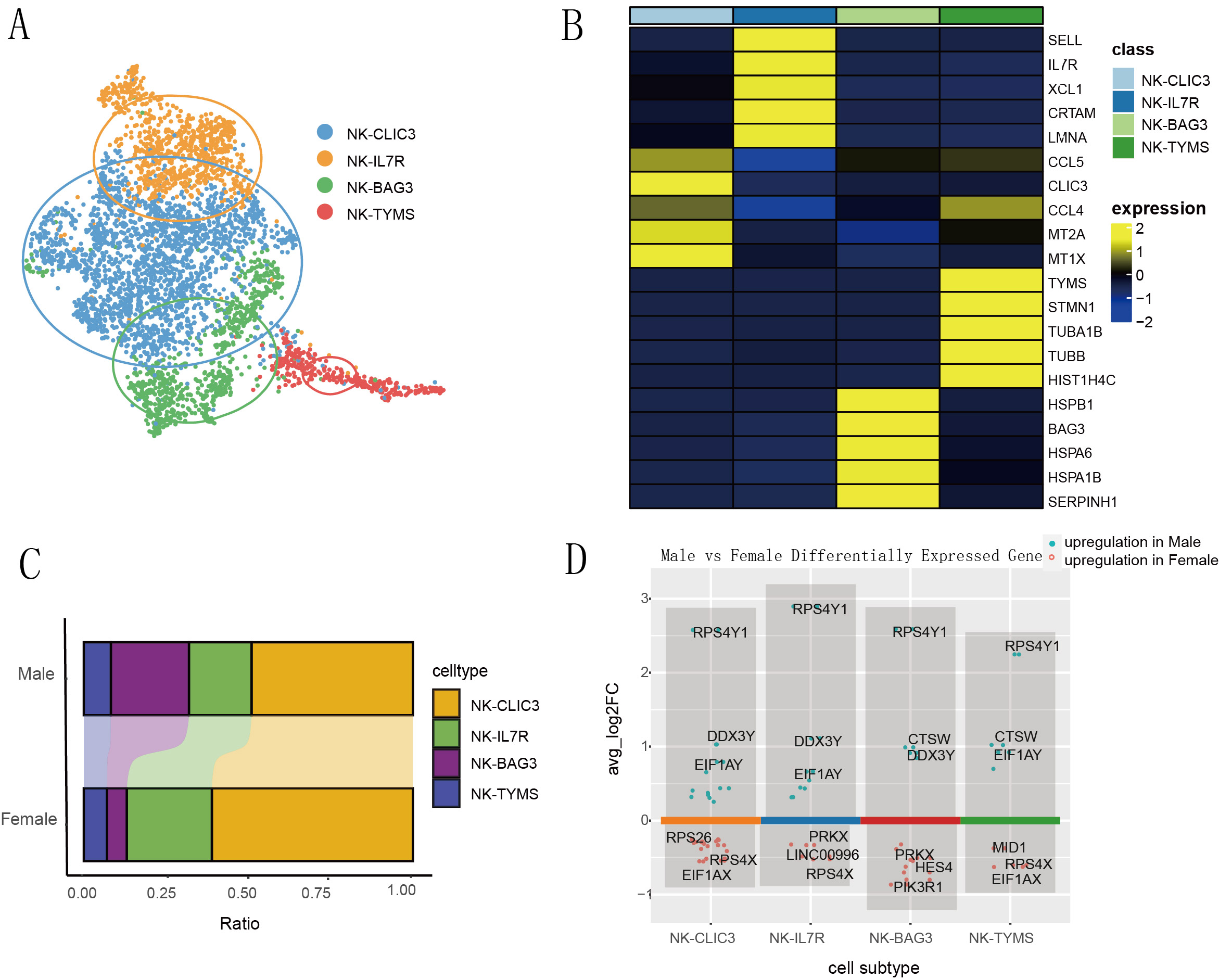

Supplement: Supplementary file 15 — Supplementary Material 15 [file 13293_2024_598_MOESM15_ESM.jpg]
