# Supplementary material for: Sex disparities revealed by single-cell and bulk sequencing and their impacts on the efficacy of immunotherapy in esophageal cancer
Source: Biol Sex Differ. 2024 Mar 15;15:22. doi: 10.1186/s13293-024-00598-z (PMC10941500; doi:10.1186/s13293-024-00598-z)
Supplement: Supplementary file 8 — Supplementary Material 8 [file 13293_2024_598_MOESM8_ESM.docx]

**Suppl Table 1. Baseline Characteristics of the randomized phase 3 trials included in clinical analysis.**

| **Study** | **Masking** | **Line of Treatment** | **Treatment agent** | **No. of Patients** | **Male/Female** | **Median follow-up (months)** | **OS (HR, 95% CI)** | **Jadad Score** |
| --- | --- | --- | --- | --- | --- | --- | --- | --- |
| ATTRACTION-3^1^ | Open-label | 2 | Nivolumab | 210 | 179/31 | 10.5 | 0.79 (0.64-0.97) | 4 |
|  |  |  | Chemotherapy | 209 | 185/24 | 8.0 | Reference |  |
| CheckMate 648^2^ | Open-label | 1 | Nivolumab + chemotherapy | 321 | 253/68 | >13.0 | 0.74 (0.58-0.96) | 4 |
|  |  |  | Nivolumab + ipilimumab | 325 | 269/56 |  | 0.78 (0.62-0.98) |  |
|  |  |  | Chemotherapy | 324 | 275/49 |  | Reference |  |
| KEYNOTE-181*^, 3^ | Open-label | 2 | Pembrolizumab | 107 | 191/31 | 7.1 | 0.69 (0.52-0.93) | 4 |
|  |  |  | Chemotherapy | 115 |  | 6.9 | Reference |  |
| KEYNOTE-590^4^ | Double-Blind | 1 | Pembrolizumab + chemotherapy | 373 | 306/67 | 22.6 | 0.73 (0.62-0.86) | 5 |
|  |  |  | Chemotherapy | 376 | 319/57 |  | Reference |  |

CI, confidence interval; HR, hazard ratio; OS, overall survival

* Patients that were PD-L1 positive were included because FDA approved the application of pembrolizumab monotherapy only in this population.

**Suppl Table 2. Baseline features of the datasets included in the bulk analysis.**

| **Datasets** | **Publication year** | **Samples** | **Male/Female** | **Raw SNVs** | **Verified SNVs** | **Detection methods** |
| --- | --- | --- | --- | --- | --- | --- |
| PMID34413060^5^ | 2021 | 5 | 5/0 | 2104 | 2101 | Agilent SureSelect XT Human All Exon V5 Plus Regulatory |
| PMID28548104^6^ | 2017 | 92 | 81/11 | 6797 | 6764 | WGS |
| PMID32974170^7^ | 2020 | 21 | 10/11 | 8790 | 7300 | Agilent SureSelectXT Human All Exon V5 |
| PMID30975989^8^ | 2019 | 39 | 28/11 | 7616 | 7593 | Agilent SureSelect Human All Exon V6 |
| PMID27058444^9^ | 2016 | 67 | 46/21 | 9918 | 9853 | Agilent Sure-Select Human All Exon V4 plus UTRs |
| PMID25839328^10^ | 2015 | 104 | 104/0 | 10149 | 10134 | NimbleGen SeqCap EZ Exome (44M) |
| PMID25151357^11^ | 2014 | 113 | 94/19 | 12022 | 11990 | Agilent SureSelect Human All Exon V4 |
| PMID34263978 ^12^ | 2021 | 88 | 81/7 | 12683 | 12632 | Agilent SureSelect XT Human All Exon |
| TCGA^13^ | 2020 | 94 | 80/14 | 18858 | 18781 | N.A. |
| PMID34285259^14^ | 2021 | 10 | 9/1 | 21628 | 21319 | Agilent SureSelect XT Human All Exon V6 + UTR kit |
| PMID26873401^15^ | 2016 | 144 | 126/18 | 23121 | 23059 | SureSelect Human All Exon 50Mb Kit |
| ICGC^13^ | 2020 | 140 | 101/39 | 24208 | 23962 | N.A. |
| PMID32398863^16^ | 2020 | 508 | 335/173 | 7218110 | 7210261 | WGS |

SNV, single nucleotide variant; WGS, Whole Genome Sequencing

**References:**

1. Kato K, Cho BC, Takahashi M, et al. Nivolumab versus chemotherapy in patients with advanced oesophageal squamous cell carcinoma refractory or intolerant to previous chemotherapy (ATTRACTION-3): a multicentre, randomised, open-label, phase 3 trial. *The Lancet Oncology* 2019; **20**(11): 1506-17.

2. Doki Y, Ajani JA, Kato K, et al. Nivolumab Combination Therapy in Advanced Esophageal Squamous-Cell Carcinoma. *The New England journal of medicine* 2022; **386**(5): 449-62.

3. Kojima T, Shah MA, Muro K, et al. Randomized Phase III KEYNOTE-181 Study of Pembrolizumab Versus Chemotherapy in Advanced Esophageal Cancer. *Journal of clinical oncology : official journal of the American Society of Clinical Oncology* 2020; **38**(35): 4138-48.

4. Sun JM, Shen L, Shah MA, et al. Pembrolizumab plus chemotherapy versus chemotherapy alone for first-line treatment of advanced oesophageal cancer (KEYNOTE-590): a randomised, placebo-controlled, phase 3 study. *Lancet (London, England)* 2021; **398**(10302): 759-71.

5. Hirata H, Niida A, Kakiuchi N, et al. The Evolving Genomic Landscape of Esophageal Squamous Cell Carcinoma Under Chemoradiotherapy. *Cancer research* 2021; **81**(19): 4926-38.

6. Chang J, Tan W, Ling Z, et al. Genomic analysis of oesophageal squamous-cell carcinoma identifies alcohol drinking-related mutation signature and genomic alterations. *Nature communications* 2017; **8**: 15290.

7. Mangalaparthi KK, Patel K, Khan AA, et al. Mutational Landscape of Esophageal Squamous Cell Carcinoma in an Indian Cohort. *Frontiers in oncology* 2020; **10**: 1457.

8. Yan T, Cui H, Zhou Y, et al. Multi-region sequencing unveils novel actionable targets and spatial heterogeneity in esophageal squamous cell carcinoma. *Nature communications* 2019; **10**(1): 1670.

9. Qin HD, Liao XY, Chen YB, et al. Genomic Characterization of Esophageal Squamous Cell Carcinoma Reveals Critical Genes Underlying Tumorigenesis and Poor Prognosis. *American journal of human genetics* 2016; **98**(4): 709-27.

10. Zhang L, Zhou Y, Cheng C, et al. Genomic analyses reveal mutational signatures and frequently altered genes in esophageal squamous cell carcinoma. *American journal of human genetics* 2015; **96**(4): 597-611.

11. Gao YB, Chen ZL, Li JG, et al. Genetic landscape of esophageal squamous cell carcinoma. *Nature genetics* 2014; **46**(10): 1097-102.

12. Takemoto A, Tanimoto K, Mori S, et al. Integrative genome-wide analyses reveal the transcriptional aberrations in Japanese esophageal squamous cell carcinoma. *Cancer science* 2021; **112**(10): 4377-92.

13. Pan-cancer analysis of whole genomes. *Nature* 2020; **578**(7793): 82-93.

14. Erkizan HV, Sukhadia S, Natarajan TG, et al. Exome sequencing identifies novel somatic variants in African American esophageal squamous cell carcinoma. *Scientific reports* 2021; **11**(1): 14814.

15. Sawada G, Niida A, Uchi R, et al. Genomic Landscape of Esophageal Squamous Cell Carcinoma in a Japanese Population. *Gastroenterology* 2016; **150**(5): 1171-82.

16. Cui Y, Chen H, Xi R, et al. Whole-genome sequencing of 508 patients identifies key molecular features associated with poor prognosis in esophageal squamous cell carcinoma. *Cell research* 2020; **30**(10): 902-13.
