# Supplementary material for: Sex disparities revealed by single-cell and bulk sequencing and their impacts on the efficacy of immunotherapy in esophageal cancer
Source: Biol Sex Differ. 2024 Mar 15;15:22. doi: 10.1186/s13293-024-00598-z (PMC10941500; doi:10.1186/s13293-024-00598-z)
Supplement: Supplementary file 1 — Supplementary Material 1 [file 13293_2024_598_MOESM1_ESM.docx]

Dear Dr. Joshua Rubin:

Please find enclosed manuscript entitled “Sex disparities revealed by single-cell and bulk sequencing data and their impacts on the efficacy of immunotherapy in esophageal cancer”, which we would like to re-submit for publication in Biology of Sex Differences.

We thank you for your kindly advices and thank the reviewers for their careful examination of the manuscript and insightful comments. We have revised our paper according to their suggestions and addressed all the concerns in details. We sincerely hope that the revised manuscript is acceptable for publication.

We shall look forward to hearing from you.

Yours Sincerely,

Prof. Bin Zhao

Reviewer reports:

Reviewer #1: Yan et.al explored the genomic and cellular sex disparities in esophageal cancer through a comprehensive and systematic analysis, and revealed the association between sex and the efficacy of immunotherapy. Generally, this study provides valuable insight into the center of the new wave of cancer immunotherapy. The paper is well written and organized. The introduction explains in a clear and coherent manner the background of this study. The authors made tremendous efforts to collect, process, and analyze the eligible dataset. The results are provocative, helpful and insightful. There are some points that the authors should address to improve the quality of the manuscript.
1. The authors conducted non-negative matrix factorization analysis of mutational signatures to extracted mutations pattern in EC. How many SBS were there? Could the authors provide the whole mutation patterns?

Totally, the frequencies of 78 mutational signatures were compared between male and female EC patients. We added one more figure (Suppl. Figure 3) to illustrate all the investigated mutation patterns. We revised the manuscript accordingly (line 271-272)

2.Over 130 altered molecular pathways were different between male and female. However, none of them were in the top 5 common altered reactome pathways illustrated in Figure 3. Hence, I suggested the authors put Figure 3 in the supplementary materials.

As suggested, in the revised manuscript, figure 3 were renamed as suppl. Figure 4 in the supplementary materials section.

3.The authors constructed a sex-related signature to predict the survival of immunotherapy, and its performances is more powerful than TMB. I wonder how did the authors define high TMB and low TMB, and why?

In the present study, the cut-off value for high TMB and low TMB was defines as the median value of the whole TMB in each cohort. Because in such circumstances, the whole patient population could be divided into two subgroups with similar size. Additionally, due to the potential bias and/or errors in the original study, we calculated the TMB in each cohort separately to make sure our signature was better than TMB in every cohort.

4.All identified HLA-II antigens were enriched in female, but no male bias at all? How many patients were analyzed here?

Yes, based on our analysis, we did not find any HLA-II antigens that were enriched in males. As the reviewer implied, it might be because only limited number of patients were included in this analysis. Here, a total of 183 patients were included in our analysis. We revised the manuscript accordingly (line 334).

5.It is very interesting that the exhausted CD8+ T cells were so enriched in male EC patients, the authors should explore the potential underlying mechanism between sex and CD8+ T cells in a more in-depth manner.

We totally agree with the reviewer on this point. As suggested, we added one whole paragraph (line 516-530) on the potential molecular mechanisms of the sex disparity in CD8+ T cells. We thank the reviewer very much for his/her kindly advise.

This thorough and systematic study is indeed commendable, and with a few enhancements, it could offer a substantial contribution to the field. I look forward to the revisions and further development of this manuscript. 

Reviewer #2: In this manuscript, Yan et al. have conducted a metanalysis of randomized ICI trials to investigate whether there is sex dimorphism in response to immunotherapies. This study reveals crucial intrinsic and extrinsic differences of EC tumors including several mutations, enriched pathways, and immune cell subsets. Although the study is of high quality, concerns regarding results and conclusions still need to be addressed. Here are some points that need addressing:
-  M&m why information obtained from metastatic sites was excluded?
Current evidence suggested that the sex-based dimorphism in the efficacies of immunotherapy was associated with tumor type. For example, women with advanced lung cancer showed more survival benefit than man from the combination of immunotherapy and chemotherapy. In contrast, men had higher overall survival rates compared to women in melanoma. We were afraid that tumors with metastatic sites might lead some bias into our study, accordingly information derived from metastatic sites were not included here.

-  Please reflect on the biased inclusion of women ca. only 17%, most likely because of the biased incidence rate. Include such information in the introduction or the result section.
We thank the reviewer very much for his/her insightful suggestion. We revised our paper by adding the incidence bias globally in Introduction Section (line 95-96), and the most of patients recruited in phase 3 trials were male (n=1977, 84%) (line 221). Please check the manuscript for details.

-  Analysis revealed that males favor immunotherapy, while women favor chemotherapy, could this be explained by specific mutations in the different sexes? Such association should be investigated and computationally validated by therapy prediction analysis and test cohorts.
We totally agree with the reviewer on this point. In fact, we conducted such analysis on all the different mutant genes, SBS and pathways that showed differences between male and female. However, we cannot find any solid associations between any single mutation/SBS/pathway and outcomes in EC. We suspected that, in EC, any single feature was insufficient to impact the whole landscape of anti-cancer immune response. That is why we developed a sex-based signature in our study to predict the outcomes.

-  Gene network analysis is highly recommended to predict the affected pathways by the mutated genes in each sex.  
We thank the reviewer very much for his/her insightful comment. As suggested, we examined the gene network affected by the mutant genes via GO and KEGG analysis. We presented them in figure 2 and suppl. Figure 2, and revised the manuscript accordingly.

-  Figure 2 is not complete; the lower panel is cut and no labels to distinguish males from females.
We feel very sorry for that and replaced it with a new one in the revised paper. The mutation frequencies in male (numbers in blue) and female (numbers in red) of were showed in every SBS panels and were annotated in the figure legends.

-  Figure 3, the scope of the manuscript is the sex dimorphism in patients receiving ICI vs. ctrl, thus all figures should focus on and show the differences/similarities between the two sexes.
This comment was similar with comment 2 from reviewer No. 1. As suggested, we put the original figure 3 in the supplementary materials as suppl. Figure 4. Additionally, we replaced it with one new figure to show the differences between male and female.

-  Did the frequency of different HLA-II alleles affect the OS in response to ICI?
The reviewer has a good point here. In the present study, we investigated the frequencies of different HLA-II alleles in male and female. It is reasonable to check whether these differences could affect the outcomes of immunotherapy, like what we did on mutation/SBS/pathway/ cellular subpopulations. However, there is no available data regarding the association between HLA-II and OS in immunotherapy even we made tremendous efforts to find. Hence, we cannot conduct such analysis here. We understand it is a very interesting and important issue in exploring the mechanisms of cancer immunotherapy. Accordingly, we are starting to collect our own samples right now, and hope we can address this issue in our next paper.

-  Figure 5, The figure panels must be described in chronological order.

Done.

-  Figure 6, The abundance of CD8-Tex in responses to ICI, what about naïve CD8 T cells, can they predict a lack of responses?

We thank the reviewer very much for his/her insightful comment. Indeed, we examined the predictive value of naïve CD8 T cells as a biomarker in immunotherapy. As shown in suppl. Figure 6, the performances of naïve CD8 T cells were not so powerful as CD8-Tex in predicting the efficacies of immunotherapy in three cohorts (We cannot find any naïve CD8 T cells in the fourth cohort). We revised our manuscript accordingly (line 401-403).

-  The data shows that exhausted CD8 T cells are accumulated in men and naïve CD8 T cells in women, efforts to explain why antigen-experienced T cells are present in men but not in women should be made.

This comment was similar with the fifth comment of reviewer No.1. we added one whole paragraph (line 516-530) on the potential molecular mechanisms of the sex disparity in CD8+ T cells. Generally speaking, current theory suggested that androgen receptor could mediated the exhausted state of CD8+ T cells through several signaling pathways.

-  There is a big difference between males and females in C2, this population needs attention and further investigations.
We thank the reviewer very much for pointing this out. Yes, the proportion of one specific subgroup of NK cells showed robust sex disparities. Interestingly, the featured maker gene in this subtype was *BAG3*, a very common label for exhausted T cells. We totally agree with the reviewer that further investigations were needed to disclose the underlying mechanisms between this specific cell population and the efficacy of cancer immunotherapy. We revise the manuscript accordingly (line 414-418).
